# Transmembrane Serine Protease 2 and Proteolytic Activation of the Epithelial Sodium Channel in Mouse Kidney

**DOI:** 10.1681/ASN.0000000521

**Published:** 2024-10-23

**Authors:** Florian Sure, Sara Afonso, Daniel Essigke, Paul Schmidt, M. Zaher Kalo, Viatcheslav Nesterov, Alicia Kißler, Marko Bertog, Ralf Rinke, Sabine Wittmann, Katharina A.E. Broeker, Thomas Gramberg, Ferruh Artunc, Christoph Korbmacher, Alexandr V. Ilyaskin

**Affiliations:** 1 Friedrich-Alexander-Universität Erlangen-Nürnberg, Institute of Cellular and Molecular Physiology, Erlangen, Germany; 2 Division of Endocrinology, Diabetology and Nephrology, Department of Internal Medicine, University Hospital Tübingen, Tübingen, Germany; 3 Institute of Diabetes Research and Metabolic Diseases (IDM) of the Helmholtz Center Munich at the University Tübingen, Tübingen, Germany; 4 German Center for Diabetes Research (DZD) at the University Tübingen, Tübingen, Germany; 5 Friedrich-Alexander-Universität Erlangen-Nürnberg, Institute of Clinical and Molecular Virology, Universitätsklinikum Erlangen, Erlangen, Germany; 6 Institute of Physiology, University of Regensburg, Regensburg, Germany

**Keywords:** aldosterone, BP, cell and transport physiology, collecting ducts, electrophysiology, ENaC, epithelial sodium channel, ion transport, transgenic mouse, kidney biology and physiology

## Abstract

**Key Points:**

- Proteolytic activation of the epithelial sodium channel (ENaC) was compromised by transmembrane serine protease 2 deficiency in murine cortical collecting duct cells and native mouse kidney.
- To compensate for impaired ENaC activation, rise in plasma aldosterone in response to low-salt diet was enhanced in *Tmprss2*^−/−^ mice.
- Transmembrane serine protease 2 may be a potential drug target to limit proteolytic ENaC activation in disorders with increased renal ENaC activity.

**Background:**

The renal epithelial sodium channel (ENaC) is essential for sodium balance and BP control. ENaC undergoes complex proteolytic activation by not yet clearly identified tubular proteases. Here, we examined a potential role of transmembrane serine protease 2 (TMPRSS2).

**Methods:**

Murine ENaC and TMPRSS2 were (co)expressed in *Xenopus laevis* oocytes. ENaC cleavage and function were studied in TMPRSS2-deficient murine cortical collecting duct (mCCD_cl1_) cells and TMPRSS2-knockout (*Tmprss2*^*−/−*^) mice. Short-circuit currents (*I*_SC_) were measured to assess ENaC-mediated transepithelial sodium transport of mCCD_cl1_ cells. The mCCD_cl1_ cell transcriptome was studied using RNA sequencing. The effect of low-sodium diet with or without high potassium were compared in *Tmprss2*^*−/−*^ and wild-type mice using metabolic cages. ENaC-mediated whole-cell currents were recorded from microdissected tubules of *Tmprss2*^*−/−*^ and wild-type mice.

**Results:**

In oocytes, coexpression of murine TMPRSS2 and ENaC resulted in fully cleaved *γ*-ENaC and approximately two-fold stimulation of ENaC currents. High baseline expression of TMPRSS2 was detected in mCCD_cl1_ cells without a stimulatory effect of aldosterone on its function or transcription. TMPRSS2 knockout in mCCD_cl1_ cells compromised *γ*-ENaC cleavage and reduced baseline and aldosterone-stimulated *I*_SC_, which could be rescued by chymotrypsin. A compensatory transcriptional upregulation of other proteases was not observed. *Tmprss2*^−/−^ mice kept on standard diet exhibited no apparent phenotype, but renal *γ*-ENaC cleavage was altered. In response to a low-salt diet, particularly with high potassium intake, *Tmprss2*^−/−^ mice increased plasma aldosterone significantly more than wild-type mice to achieve a similar reduction of renal sodium excretion. Importantly, the stimulatory effect of trypsin on renal tubular ENaC currents was much more pronounced in *Tmprss2*^−/−^ mice than that in wild-type mice. This indicated the presence of incompletely cleaved and less active channels at the cell surface of TMPRSS2-deficient tubular epithelial cells.

**Conclusions:**

TMPRSS2 contributes to proteolytic ENaC activation in mouse kidney *in vivo*.

## Visual Abstract

**Figure:**
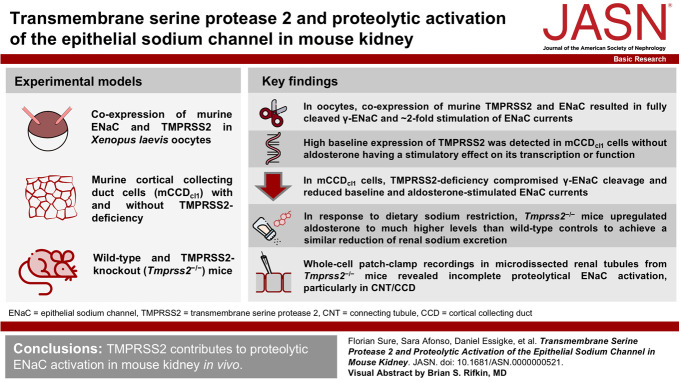

## Introduction

The epithelial sodium channel (ENaC) is a heterotrimeric ion channel that consists of an *α*, *β*, and *γ* subunit and belongs to the ENaC/degenerin family of ion channels.^1–3^ ENaC provides the rate-limiting step for transepithelial sodium absorption in several epithelia. Among these is the distal nephron comprising the late distal convoluted tubule (DCT2), the connecting tubule (CNT), and the cortical collecting duct (CCD). Precise ENaC regulation in the distal nephron is essential for adjusting renal sodium excretion to oral intake and, hence, for maintaining sodium homeostasis, extracellular volume, and BP.^4,5^ Aldosterone plays a key role in hormonal ENaC stimulation, in particular in the CNT/CCD transition zone, where ENaC activity is strictly aldosterone-dependent. By contrast, ENaC activity is largely aldosterone-independent in the DCT2/CNT region,^6–9^ albeit dependent on the mineralocorticoid receptor.^10,11^

It is well established that ENaC requires specific proteolytic processing to become an active channel.^12–15^ Proteolytic cleavage removes autoinhibitory peptide fragments from the extracellular loops of *α*- and *γ*-ENaC.^16–18^ According to the currently accepted paradigm, the serine protease furin and/or related furin-like proprotein convertases target three cleavage sites (two in *α*- and one in *γ*-ENaC) during channel maturation in the intracellular biosynthetic pathway.^13,19^ Importantly, a final cleavage event in *γ*-ENaC is required to achieve full channel activation.^20,21^ Relevant proteases involved in this pivotal last cleavage event in *γ*-ENaC in the kidney remain elusive.^22,23^ These may include membrane-anchored proteases expressed by tubular cells or soluble plasma proteases aberrantly filtered in disease states (*e.g*., nephrotic syndrome).^24–27^

Recently, we have demonstrated that transmembrane serine protease 2 (TMPRSS2), a membrane-anchored serine protease with trypsin-like substrate specificity, proteolytically activates human ENaC in a heterologous expression system and H441 airway epithelial cells.^28^ TMPRSS2 is highly expressed in the renal distal nephron, where it may functionally interact with ENaC.^29–34^ In addition, TMPRSS2 could interact with ENaC through the secretion of its catalytic domain or through urinary microvesicles.^35–37^ Therefore, we hypothesized that TMPRSS2 may contribute to proteolytic ENaC activation in the kidney. To test this hypothesis, we explored the effect of TMPRSS2 deficiency on renal ENaC cleavage and function in murine model systems.

## Methods

More methodological details can be found in the Supplemental Material.

### Two-Electrode Voltage-Clamp Experiments in *Xenopus laevis* Oocytes

Isolation of oocytes and two-electrode voltage-clamp experiments were performed essentially as described previously.^28,38,39^

### TMPRSS2 Knockout in Murine Cortical Collecting Duct Cells, Ussing Chamber Measurements, and RNA Sequencing

The murine cortical collecting duct (mCCD_cl1_) cell line^40^ was kindly provided by Bernard C. Rossier and Edith Hummler (Université de Lausanne, Switzerland). TMPRSS2-knockout (TMPRSS2-ko) and nontargeting control mCCD_cl1_ cells were generated using clustered regularly interspaced short palindromic repeats /Cas9.^41,42^ Equivalent short-circuit current (*I*_SC_) measurements were performed on mCCD_cl1_ cells grown on permeable supports.^43–45^ mRNA was isolated using the NucleoSpin RNA kit (Machery-Nagel), and RNA sequencing (RNA-seq) was performed at the Next Generation Sequencing Core Unit (Institute of Human Genetics, FAU Erlangen-Nürnberg).

### Mouse Studies

An established TMPRSS2-ko mouse model (global constitutive TMPRSS2-ko; background: C57BL/6J)^46^ was obtained from Jackson Laboratories (B6.129-*Tmprss2*^tm1Psn^/J, JAX stock 026196). The effects of different diets were studied in metabolic cages. Plasma aldosterone was measured using an ELISA kit (IBL, Hamburg, Germany).

### Immunoblotting

*γ*-ENaC was detected using an established antibody (Stressmarq; catalog no.: SPC-405) after deglycosylation (PNGase F, New England BioLabs).^47^ For TMPRSS2 detection, a commercially available antibody^37^ was used (EMD Millipore Corp.; clone P5H9-A3; catalog no.: MABF2158) and validated (Supplemental Figure 1).

### RNAscope Technology and Immunostainings

*Tmprss2* mRNA was detected in mouse kidney using the RNAscope Multiplex Fluorescent v2 kit (Advanced Cell Diagnostics, Cat. No 323100) according to the manufacturer's protocol.^48,49^ To detect *β*- or *γ-*ENaC, a polyclonal rabbit antibody directed against mouse *β*-ENaC^44^ or rat *γ-*ENaC (see above) was used, respectively.

### Preparation of Renal Tubules and Electrophysiology

Tubules were prepared and whole-cell patch-clamp recordings were performed essentially as described previously.^6,7,10,50^ Two tubular regions were distinguished according to morphological criteria^6^: (*1*) DCT2 and initial CNT (DCT2/CNT) and (*2*) late CNT and initial CCD (CNT/CCD).

### Statistical Methods

Data are presented as mean±SEM. Normal distribution of data were assessed using the D'Agostino–Pearson omnibus or Shapiro–Wilk test. Statistical significance was assessed using appropriate tests as indicated in figure legends.

## Results

### Coexpression of Mouse TMPRSS2 Increased Mouse ENaC Currents in *Xenopus laevis* Oocytes

To test whether murine TMPRSS2 proteolytically activates murine ENaC, we expressed murine *αβγ*-ENaC in *Xenopus laevis* oocytes with or without coexpression of murine TMPRSS2. In oocytes coinjected with 0.1 ng/subunit ENaC complementary RNA and TMPRSS2 complementary RNA (0.2 ng), baseline amiloride-sensitive currents were about twice as high as in oocytes expressing ENaC alone (Supplemental Figure 2, A–C). Importantly, ENaC currents were not further stimulated by chymotrypsin application, unlike in oocytes expressing ENaC alone (Supplemental Figure 2, A–D). Consistent with these functional results, we demonstrated that TMPRSS2 coexpression converted partially cleaved *γ*-ENaC (approximately 60 kDa) at the cell surface into its fully cleaved active form (approximately 55 kDa) (Supplemental Figure 2E). Thus, using murine orthologs, we confirmed that TMPRSS2 activates ENaC by γ-ENaC cleavage.

### TMPRSS2 Contributed to Proteolytic ENaC Activation in mCCD_cl1_ Cells

**Figure 1:**
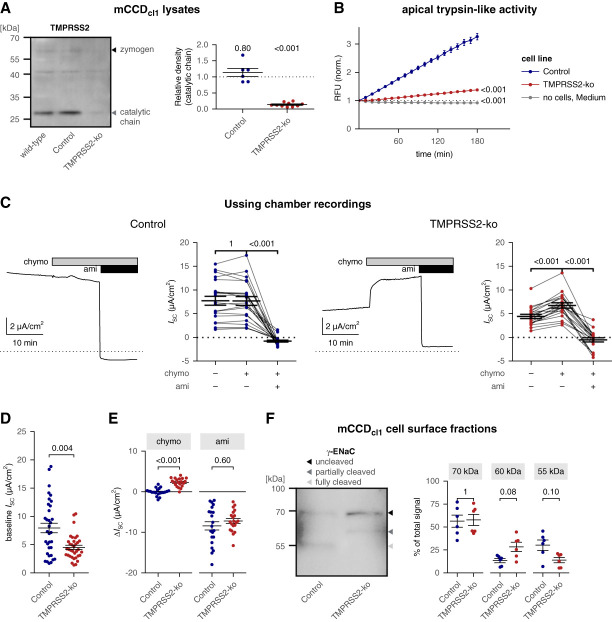
**TMPRSS2 contributed to proteolytic ENaC activation in mCCD**_**cl1**_ **cells.** (A) Left: representative Western blot analysis of whole-cell mCCD_cl1_ lysates to assess endogenous expression of TMPRSS2 in wild-type, nontargeting control (Control), and TMPRSS2-ko cells. Gray and black arrowheads indicate TMPRSS2 in its activated (catalytic chain, approximately 26 kDa) or zymogen form (approximately 60 kDa), respectively. Similar protein loading in all lanes was confirmed using Ponceau S total protein staining (Supplemental Figure 3). Right: densitometric evaluation of TMPRSS2 expression from similar blots as shown in the left. In each blot, the density value of the approximately 26 kDa band was normalized to the corresponding density value from wild-type cells. The dotted line indicates a normalized density value of one (no effect). Mean±SEM and data points for individual samples are shown; *n*=6–10, Two-sided one-sample *t* test of log-transformed values with Bonferroni correction for multiple testing. (B) Progress curves of trypsin-like proteolytic activity in medium taken from the apical compartment of nontargeting control (blue, *n*=42) or TMPRSS2-ko mCCD_cl1_ cells (red, *n*=60) are shown (mean±SEM). Freshly prepared medium served as control (gray, *n*=54). In each sample, the recorded RFU values were normalized to the RFU value at the beginning of the measurement. A dotted line indicates a relative effect of one (no change). The Kruskal–Wallis test (*P* value < 0.001) with Dunn's multiple comparisons test of log-transformed values was used to calculate *P* values for comparisons with the RFU at time point 180 minutes obtained in control cells. (C) Representative equivalent short-circuit current (*I*_SC_) traces recorded from nontargeting control (left, *n*=21) or TMPRSS2-ko mCCD_cl1_ cells (right, *n*=20) are shown. Chymotrypsin (chymo, 20 *µ*g/ml) and amiloride (ami, 10 *µ*M) were present in the apical bath solution as indicated by gray and black bars, respectively. The dotted lines indicate zero current levels. Summary data from similar experiments are shown to the right of the representative traces. *I*_SC_ values were obtained immediately before application of chymotrypsin or amiloride and at the end of the experiment. Values obtained in the same measurement are connected with a line. Mean±SEM and data points for individual measurement are shown. ANOVA (*P* values: <0.001 for control; <0.001 for TMPRSS2-ko) with the Bonferroni *post hoc* test. (D) Summary data from the same experiments as shown in (C) and in Supplemental Figure 6. Baseline *I*_SC_ at the beginning of the experiment. Mean±SEM and data points for individual measurements are shown. Two-sided unpaired Wilcoxon-signed rank test. (E) Summary data from the same experiments as shown in (C). The effect of chymotrypsin (chymo) on *I*_SC_ (Δ*I*_SC_) was calculated by subtracting the *I*_SC_ value measured immediately before chymotrypsin application from the current level reached in the presence of chymotrypsin immediately before amiloride application. The effect of amiloride (ami) on *I*_SC_ (Δ*I*_SC_) was calculated by subtracting the *I*_SC_ value measured immediately before amiloride application from the current level reached at the end of the recording. Mean±SEM and data points for individual measurements are shown. A two-sided unpaired *t* test with Bonferroni correction for multiple testing. (F) Left: representative Western blot showing cell surface expression of *γ*-ENaC in mCCD_cl1_ cells. Uncleaved (approximately 70 kDa), partially cleaved (approximately 60 kDa), and fully cleaved (approximately 55 kDa) *γ*-ENaC are indicated by black, dark gray, and light gray arrowheads, respectively. Right: densitometric evaluation of the Western blot shown in the left and additional blots shown in Supplemental Figure 8. The densitometric signal of uncleaved, partially cleaved, and fully cleaved *γ*-ENaC was normalized to the total signal of all three bands. Mean±SEM and data points for individual Western blots are shown (*n*=6). A two-sided unpaired *t* test with Bonferroni correction for multiple testing. ENaC, epithelial sodium channel; mCCD_cl1_, murine cortical collecting duct cell line; RFU, relative fluorescent unit; TMPRSS2, transmembrane serine protease 2; TMPRSS2-ko, transmembrane serine protease 2–knockout.

To investigate whether TMPRSS2 is involved in proteolytic ENaC activation in mCCD_cl1_ cells, we generated TMPRSS2-ko cells. Successful TMPRSS2-ko was confirmed using Western blot analysis (Figure 1A and Supplemental Figure 3). TMPRSS2-ko mCCD_cl1_ cells formed tight epithelial monolayers but had a significantly lower transepithelial resistance compared with control mCCD_cl1_ cells (Supplemental Figure 4A). However, overall epithelial monolayer integrity was preserved, as evidenced by immunofluorescence staining for the tight junction marker zona occludens protein 1 (Supplemental Figure 4B).

Using a fluorogenic substrate assay, we assessed trypsin-like proteolytic activity in the apical medium collected from mCCD_cl1_ cells (Figure 1B). A high degree of proteolytic activity was detected in the medium collected from control mCCD_cl1_ cells. Importantly, this proteolytic activity was significantly reduced in the medium collected from TMPRSS2-ko cells. In line with this, using protein precipitation, we detected the catalytic domain of TMPRSS2 in the apical medium of control but not of TMPRSS2-deficient cells (Supplemental Figure 5).

Reduced endogenous protease activity may impair ENaC cleavage and activation in TMPRSS2-deficient mCCD_cl1_ cells. To investigate this, we assessed ENaC-mediated transepithelial transport by *I*_SC_ measurements. Average baseline *I*_SC_ values were higher in control than in TMPRSS2-deficient cells (Figure 1, C and D). Applying chymotrypsin to the apical bath solution did not significantly alter *I*_SC_ in control cells. This indicated that endogenous proteases were sufficient for full proteolytic ENaC activation at the cell surface. By contrast, in TMPRSS2-ko mCCD_cl1_ cells, apical application of chymotrypsin substantially stimulated *I*_SC_ by 2.3±0.2 *µ*A/cm^2^, to a level similar to baseline *I*_SC_ of control cells (Figure 1, C and E). Thus, in TMPRSS2-deficient cells, proteolytic ENaC activation at the cell surface was incomplete. In the presence of amiloride, chymotrypsin failed to stimulate *I*_SC_ in mCCD_cl1_ cells with TMPRSS2 deficiency (Supplemental Figure 6). Furthermore, we demonstrated that apical application of aprotinin, a broad-spectrum serine protease inhibitor, reduced *I*_SC_ in control but not in TMPRSS2-deficient mCCD_cl1_ cells. The inhibitory effect of aprotinin on *I*_SC_ in control mCCD_cl1_ cells could be rescued by chymotrypsin (Supplemental Figure 7). These results demonstrated a substantial contribution of apical TMPRSS2 activity to proteolytic ENaC activation in mCCD_cl1_ cells, whereas the role of other proteases seemed to be negligible. The finding that proteolytic ENaC activation was incomplete in TMPRSS2-deficient mCCD_cl1_ cells was confirmed by Western blot detection of *γ*-ENaC cleavage fragments in the apical membrane of these cells. Indeed, compared with control cells, the fraction of fully cleaved cell surface *γ*-ENaC was decreased in TMPRSS2-deficient mCCD_cl1_ cells, whereas the partially cleaved *γ*-ENaC fraction was increased (Figure 1F and Supplemental Figure 8).

Taken together, these results indicated that endogenously expressed TMPRSS2 contributed to proteolytic *γ*-ENaC processing and channel activation in mCCD_cl1_ cells.

### TMPRSS2-ko Reduced the Stimulatory Effect of Aldosterone in mCCD_cl1_ Cells

**Figure 2:**
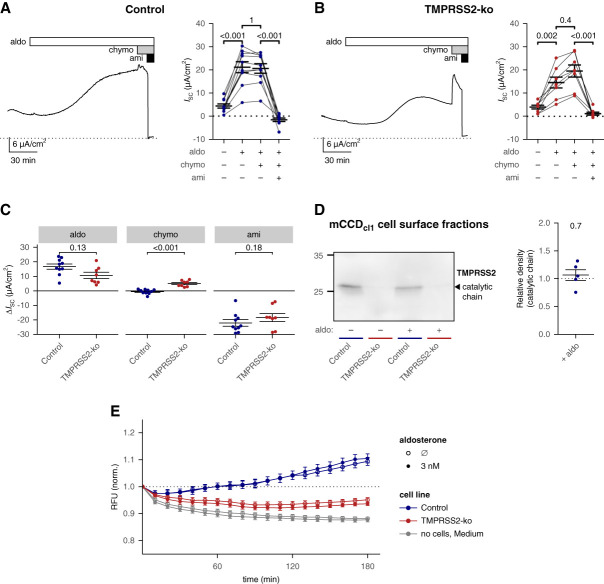
**TMPRSS2-ko reduced the stimulatory effect of aldosterone in mCCD**_**cl1**_ **cells.** (A and B) Left: representative equivalent short-circuit current (*I*_SC_) recordings from nontargeting control (A) or TMPRSS2-ko mCCD_cl1_ cells (B) are shown. Aldosterone (aldo, 3 nM, apical and basolateral), chymotrypsin (chymo, 20 *µ*g/ml, apical), and amiloride (ami, 10 *µ*M, apical) were present in the bath solution as indicated by white, gray, and black bars, respectively. The dotted lines indicate zero current levels. Right*:* summary data from similar experiments as shown in the corresponding left. Values were obtained immediately before application of aldosterone, chymotrypsin, or amiloride and at the end of the experiment. Values obtained in the same measurement are connected with a line. Mean±SEM and data points for individual measurements are shown. (A) *n*=10, (B) *n*=8. ANOVA (*P* values: <0.001 [A], <0.001 [B]) with a Bonferroni *post hoc* test. (C) Summary data from the same experiments as shown in (A) and (B). Δ*I*_SC_ was calculated essentially as described in Figure 1E. Mean±SEM and data points for individual measurements are shown. A two-sided unpaired Wilcoxon signed-rank test with Bonferroni correction for multiple testing. (D) Left: Western blot analysis of the apical cell–surface fraction of mCCD_cl1_ cells to assess endogenous expression of TMPRSS2 in nontargeting control (control, blue bars) and TMPRSS2-ko (red bars) cells. A black arrowhead indicates TMPRSS2 in its activated (catalytic chain, approximately 26 kDa) form. The absence of the approximately 26 kDa TMPRSS2 band in TMPRSS2-ko cells confirmed the specificity of TMPRSS2 detection. Before harvesting, cells were maintained for 3 hours in the presence (+) or absence (−) of 3 nM aldosterone (aldo) as indicated. Right: densitometric evaluation of TMPRSS2 expression in control cells from Western blots shown in the left and an additional blot shown in Supplemental Figure 11A. In each blot, the density value of the approximately 26 kDa TMPRSS2 band obtained from aldosterone-treated mCCD_cl1_ cells was normalized to the corresponding TMPRSS2 signal obtained from vehicle-treated mCCD_cl1_ cells. The dotted line indicates a normalized density value of one (no effect). Mean±SEM and data points for individual samples are shown (*n*=5). Two-sided one-sample *t* test with log-transformed values. (E) Progress curves of trypsin-like proteolytic activity in medium taken from the apical compartment of nontargeting control (blue, *n*=42) or TMPRSS2-ko mCCD_cl1_ cells (red, *n*=60) are shown (mean±SEM). Freshly prepared medium (with or without aldosterone) served as control (gray, *n*=54). In each sample, the recorded RFU values were normalized to the RFU value at the beginning of the measurement. Cells received either standard medium (Ø aldo, open symbols) or medium supplemented with 3 nM aldosterone (closed symbols) on apical and basolateral sides. Samples were taken 3 hours after medium exchange, which probably explains lower normalized RFU values compared with Figure 1B where samples were taken after 12 hours. A dotted line indicates the relative effect of one (no change). Two-way ANOVA (*P* values: <0.001 [effect of cell type], 1 [effect of aldosterone]) of log-transformed values.

Next, we studied whether TMPRSS2 deficiency altered the stimulatory effect of aldosterone on ENaC in this cell line. In control cells, aldosterone (3 nM) increased *I*_SC_ over 2 hours from 4.3±0.9 to 21.0±2.4 *µ*A/cm^2^. Subsequent addition of chymotrypsin to the apical compartment had no significant additional stimulatory effect on *I*_SC_ (Figure 2, A and C). Thus, ENaC was fully proteolytically activated at the cell surface of aldosterone-treated cells. Exposure of TMPRSS2-deficient mCCD_cl1_ cells to aldosterone also resulted in an *I*_SC_ increase. However, the degree of stimulation (from 3.9±0.8 to 14.5±2.3 *µ*A/cm^2^) seemed to be reduced compared with control cells (Figure 2, B and C). Importantly, in aldosterone-treated TMPRSS2-deficient cells subsequent apical application of chymotrypsin caused a substantial additional rise in *I*_SC_ of 5.0±0.7 *µ*A/cm^2^ (Figure 2C), essentially rescuing the full stimulatory effect of aldosterone. Indeed, in aldosterone- and chymotrypsin-treated TMPRSS2-deficient cells, application of amiloride at the end of the experiments caused an *I*_SC_ decrease similar to that observed in control mCCD_cl1_ cells having received the same treatment. Thus, in TMPRSS2-deficient cells, the total amount of ENaC present at the cell surface after aldosterone treatment was similar to that in control cells (Figure 2C). It is noteworthy that in TMPRSS2-deficient mCCD_cl1_ cells, the stimulatory effect of chymotrypsin on *I*_SC_ after aldosterone treatment was enhanced by approximately 2.2-fold compared with that observed in the absence of aldosterone (5.0±0.7 *µ*A/cm^2^ in the presence versus 2.3±0.2 *µ*A/cm^2^ in the absence of aldosterone; *P* = 0.008, two-sided *t* test; Figures 1, C and E, and 2, B and C). This indicated that aldosterone increased ENaC at the cell surface also in TMPRSS2-deficient cells, but that proteolytic activation of newly inserted channels remained incomplete because of TMPRSS2 deficiency.

To summarize, TMPRSS2 deficiency impaired the stimulatory effect of aldosterone by partially preventing proteolytic channel activation.

### Aldosterone Did Not Upregulate TMPRSS2 Transcription, and TMPRSS2 Deficiency Was Not Associated with a Substantial Transcriptional Upregulation of Other Serine Proteases

Using RNA-seq analysis of mCCD_cl1_ cells, we demonstrated that TMPRSS2 had the highest level of mRNA expression among transmembrane serine proteases detected (Table 1 and Supplemental Figures 9, E and F, and 12C). To investigate whether aldosterone regulates TMRPSS2 expression, mCCD_cl1_ cells were treated for 2 or 24 hours with 3 nM aldosterone (Supplemental Figure 10) before RNA isolation. After a 2-hour aldosterone exposure, we observed transcriptional upregulation of known aldosterone target genes,^51^ but not of *Tmprss2* or any other serine protease (Table 1, Supplemental Figure 9C, and Supplemental Table 1). With a 24-hour aldosterone treatment, only a minor 1.09-fold transcriptional upregulation of *Tmprss2* could be detected (Table 1, Supplemental Figure 9D, and Supplemental Table 2). In line with the RNA-seq data, TMPRSS2 protein expression at the cell surface (Figure 2D and Supplemental Figure 11A), trypsin-like proteolytic activity, and the amount of TMPRSS2 in the apical medium were not significantly affected by aldosterone exposure (Figure 2E and Supplemental Figure 11B). To conclude, aldosterone had no substantial effect on expression or activity of TMPRSS2 in mCCD_cl1_ cells.

**Table 1:** Effects of aldosterone treatment and transmembrane serine protease 2 deficiency on the expression of serine proteases in murine cortical collecting duct (mCCD_cl1_) cells

Gene Name	Gene Description	Average Expression in Control (TPM)	2-h Aldo		24-h Aldo		TMPRSS2-ko	
			Fold Change	Adj. *P* Value	Fold Change	Adj. *P* Value	Fold Change	Adj. *P* Value
*Tmprss2*	Transmembrane protease, serine 2	266±25	1.03	0.95	1.09^a^	7E−3^a^	0.37^a^	<9E−99^a^
*St14*	Suppression of tumorigenicity 14 (matriptase)	110±2	1.00	1.00	1.00	1.00	0.91^a^	4E−3^a^
*Prss23*	Protease, serine 23	102±5	0.99	0.75	0.99	0.46	1.20^a^	6E−3^a^
*Furin*	Furin (subtilisin-like proprotein convertase 1)	92±2	1.00	0.98	1.00	0.92	1.00	0.90
*Prss8*	Protease, serine 8 (prostasin)	86±4	1.02	1.00	1.02	0.85	0.99	0.36

Listed are five genes encoding serine proteases with the highest expression in control cells from the RNA sequencing analyses shown in Supplemental Figures 9 and 12 and Supplemental Tables 1–3. Fold change in RNA levels after 2 hours (*n*=6) or 24 hours exposure to 3 nM of aldosterone (*n*=7) or in transmembrane serine protease 2*–*knockout cells (*n*=6) compared with respective control cells are given with adjusted *P* values. Please note that despite residual expression of altered transmembrane serine protease 2 transcripts, on protein and functional level transmembrane serine protease 2–knockout was successful as shown in Figures 1, A and B, and 2, D and E, and Supplemental Figures 5 and 11B. Adj., adjusted; TPM, transcripts per million.

a Significant changes (adj. *P* value < 0.05).

Finally, RNA-seq analysis of TMPRSS2-deficient cells provided no evidence for marked compensatory upregulation of mRNA expression of other highly expressed transmembrane serine proteases in these cells, except for a modest (approximately 20%) increase of *Prss23* transcription (Table 1, Supplemental Figure 12, and Supplemental Table 3).

### In *Tmprss2*^−/−^ Mice, Proteolytic Processing of Renal *γ*- and *α*-ENaC Was Altered

**Figure 3:**
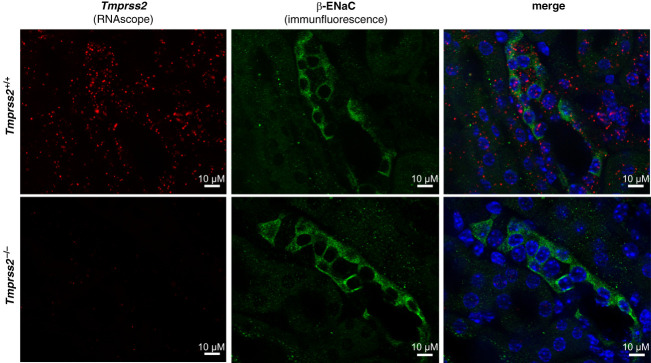
***Tmprss2* mRNA was expressed in cells positive for *β*-ENaC protein.** Representative microscopic images from mouse kidney cortex of *Tmprss2*^*+/+*^ (top row) and *Tmprss2*^*−/−*^ (bottom row) mice are shown. RNAscope staining for *Tmprss2* mRNA (left, red) and immunofluorescence staining for *β*-ENaC protein (middle, green) are merged with nuclear 4′,6-diamidin-2-phenylindol-staining (blue) in right.

To study the role of TMPRSS2 in ENaC cleavage *in vivo*, we used constitutive TMPRSS2-ko mice (*Tmprss2*^−/−^).^46^ The successful knockout of *Tmprss2* in kidneys was confirmed using RNAscope technology (Figure 3 and Supplemental Figure 13). Moreover, combining this approach with an immunofluorescence staining for *β*-ENaC, we demonstrated that *Tmprss2* mRNA was present in renal tubular cells with *β*-ENaC protein expression (Figure 3). *Tmprss2* mRNA was not restricted to ENaC-positive cells but demonstrated a ubiquitous expression pattern along the renal tubule.

**Figure 4:**
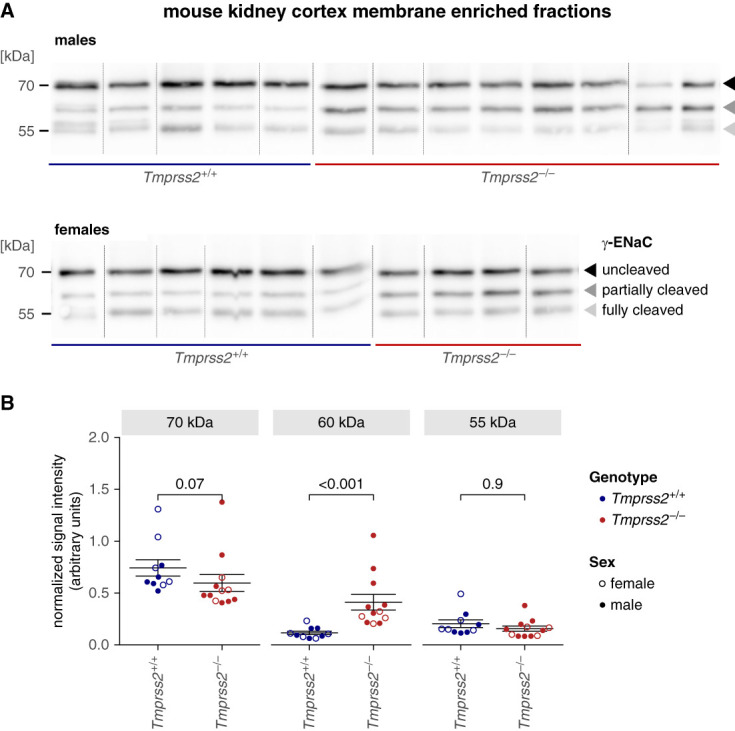
**In *Tmprss2***^***−/−***^ **mice, proteolytic processing of *γ*-ENaC was altered.** (A) Western blot analysis shows expression of endogenous PNGase-treated *γ*-ENaC in membrane-enriched fractions obtained from mouse kidney cortex lysates. Uncleaved (approximately 70 kDa), partially cleaved (approximately 60 kDa), and fully cleaved (approximately 55 kDa) *γ*-ENaC are indicated by black, dark gray, and light gray arrowheads, respectively. Vertical lines represent positions at which the original blot images were cut to reorder the lanes for clarity. Original blots are shown in Supplemental Figure 14. (B) Densitometric evaluation of similar Western blots as in (A). The densitometric signal of uncleaved, partially cleaved, and fully cleaved *γ*-ENaC in each lane was normalized to the Ponceau S total protein staining from the same lane. Mean±SEM and data points for individual Western blots are shown. Data points from female and male mice are represented with open and closed symbols, respectively. TMPRSS2 and ENaC have been reported to be modulated by sex hormones including testosterone^68^ and estrogens,^69^ respectively. However, we observed no sex-specific differences in all our analyses, and therefore, the data from male and female mice were pooled together. A two-sided Wilcoxon signed-rank test with Bonferroni correction for multiple testing.

Next, we detected *γ*- and *α*-ENaC cleavage fragments in membrane-enriched fractions obtained from kidney cortex of *Tmprss2*^+/+^ and *Tmprss2*^−/−^ mice. Similar to our observations in mCCD_cl1_ cells (Figure 1F), knockout of TMPRSS2 in mice resulted in a significant increase of the partially cleaved *γ*-ENaC fraction (Figure 4 and Supplemental Figure 14). By contrast, the signal of uncleaved or fully cleaved *γ*-ENaC in *Tmprss2*^−/−^ mice was similar to that in wild-type (*Tmprss2*^+/+^) mice. We also observed altered proteolytic processing of *α*-ENaC in kidneys from *Tmprss2*^−/−^ mice with a significantly increased portion of (furin-)cleaved *α*-ENaC at the expense of uncleaved *α*-ENaC (Supplemental Figure 15).

In summary, TMPRSS2 deficiency altered proteolytic processing of renal *γ*- and *α*-ENaC *in vivo*.

### Reduction of Renal Sodium Excretion in Response to Dietary Sodium Restriction Required Higher Plasma Aldosterone Levels in *Tmprss2*^*−/−*^ Mice than in Wild-Type Controls

**Figure 5:**
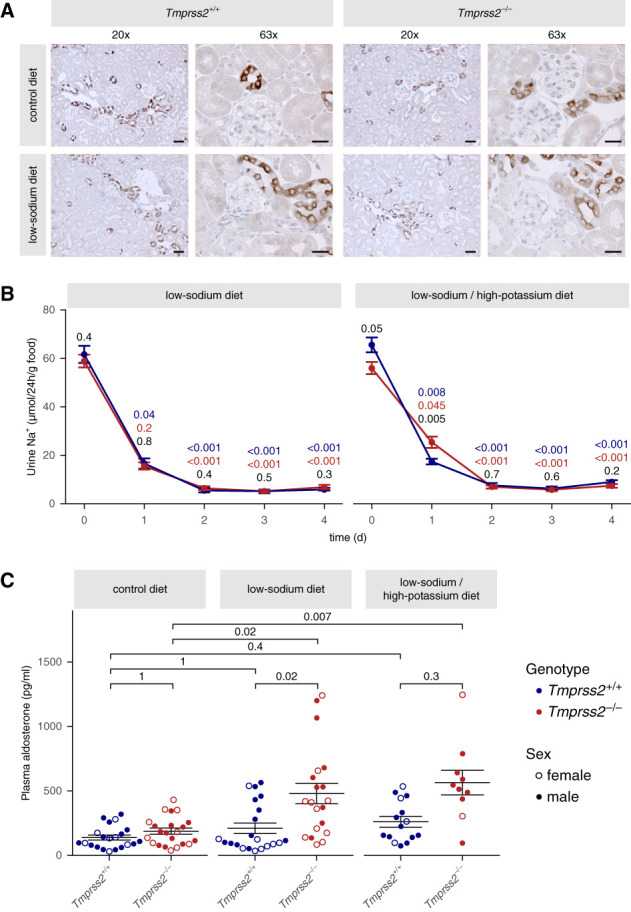
***Tmprss2***^**−/−**^ **mice required significantly higher plasma aldosterone levels than wild-type mice to maintain sodium balance under dietary sodium restriction with or without increased potassium intake.** (A) Representative immunohistochemical staining of *γ*-ENaC in fixed kidney tissue from *Tmprss2*^*+/+*^ versus *Tmprss2*^*−/−*^ mice, shown in 20× magnification (scale: 20 *µ*m) and 63× magnification (scale: 10 *µ*m). In both genotypes, there is an increased apical staining under low-sodium diet (*n*=3 each). (B) Time course of urinary sodium excretion, normalized to food intake, during dietary sodium restriction without (left) or with increased potassium intake (right). Mean±SEM (*n*=14–36) are shown. Data are pooled from both sexes. Red and blue *P* values indicate comparisons with day 0 (Friedman test with Dunn's multiple comparison test). Black *P* values indicate comparisons between genotypes (Mann–Whitney test). (C) Plasma aldosterone concentration under control diet and after 4 days of a low-sodium diet with or without increased potassium intake. Data points from female and male mice are represented with open and closed symbols, respectively. Mean±SEM and individual data points (*n*=10–22) are shown. A Kruskal–Wallis with Dunn's multiple comparison test.

Using immunohistochemistry, we found a similar tubular expression pattern of *γ*-ENaC in both genotypes (Figure 5A, top). Moreover, in *Tmprss2*^−/−^ mice, the acute natriuretic response to amiloride (Supplemental Figure 16), as well as baseline plasma Na^+^ and K^+^ concentrations (Supplemental Figure 17, A and B), were not different from those in wild-type controls. Taken together, this indicated that ENaC-mediated sodium transport was not substantially altered in *Tmprss2*^−/−^ mice under baseline conditions.

Intriguingly, both *Tmprss2*^+/+^ and *Tmprss2*^−/−^ mice responded in a similar manner to dietary sodium restriction with trafficking of ENaC to the apical membrane (Figure 5A, bottom) and an appropriate reduction of urinary sodium excretion (Figure 5B, left) to maintain sodium balance. Consistent with this finding, the acute natriuretic response to amiloride was higher in mice kept on a low-sodium diet compared with that on a control diet, but was similar in both genotypes (Supplemental Figure 16). With similar food intake and fecal Na^+^ excretion, *Tmprss2*^−/−^ and *Tmprss2*^+/+^ mice maintained similar body weight under low-salt diet (Supplemental Figures 17D and 18, A and B). In addition, in both genotypes, plasma Na^+^ and K^+^ concentrations, as well as renal K^+^ excretion, were unaffected by low-sodium diet (Supplemental Figure 17, A–C). Water intake and urine volume were slightly higher in *Tmprss2*^−/−^ mice (Supplemental Figure 18, C and D). *Tmprss2*^+/+^ and *Tmprss2*^−/−^ mice also responded in a similar manner to dietary sodium restriction in combination with an increased potassium intake (Figure 5B, right, and Supplemental Figures 19 and 20). Consistent with previously reported evidence,^47,52–55^ we detected a trend toward an increase in the cleaved *γ*-ENaC fragments in kidneys from sodium-restricted wild-type mice (Supplemental Figure 21). Interestingly, under sodium restriction, no obvious differences in proteolytic processing and overall expression of *γ*- and *α*-ENaC were observed between *Tmprss2*^+/+^ and *Tmprss2*^−/−^ mice (Supplemental Figures 21 and 22). Collectively, these data demonstrated that the ability of *Tmprss2*^*−/−*^ mice to adjust renal sodium excretion to low-sodium diet was fully preserved.

When animals were maintained on standard diet, there was a nonsignificant trend for slightly higher plasma aldosterone values in *Tmprss2*^−/−^ mice compared with *Tmprss2*^+/+^ mice (Figure 5C), averaging 187±24 and 138±19 pg/ml, respectively. After 4 days of dietary sodium restriction, there was a trend toward elevated plasma aldosterone levels (210±41 pg/ml) in *Tmprss2*^+/+^ mice (Figure 5C). This was consistent with previously reported data^38^ and seemed sufficient to downregulate renal sodium excretions adequately. Importantly, in *Tmprss2*^−/−^ mice, plasma aldosterone increased in response to dietary sodium restriction to 482±77 pg/ml, a value 2.3-fold higher than in *Tmprss2*^+/+^ mice (Figure 5C). Plasma aldosterone values reached even higher values in *Tmprss2*^−/−^ mice after 4 days of dietary sodium restriction when combined with an increased potassium intake (565±99 pg/ml, Figure 5C). This clearly indicated that in response to dietary sodium restriction, in particular in combination with increased potassium intake, *Tmprss2*^−/−^ mice needed to upregulate aldosterone to much higher levels, probably to compensate for incomplete proteolytic ENaC activation because of TMPRSS2 deficiency.

### The Stimulatory Effect of Trypsin on ENaC Activity in Microdissected Tubules Was More Pronounced in *Tmprss2*^*−/−*^ than in Wild-Type Mice

**Figure 6:**
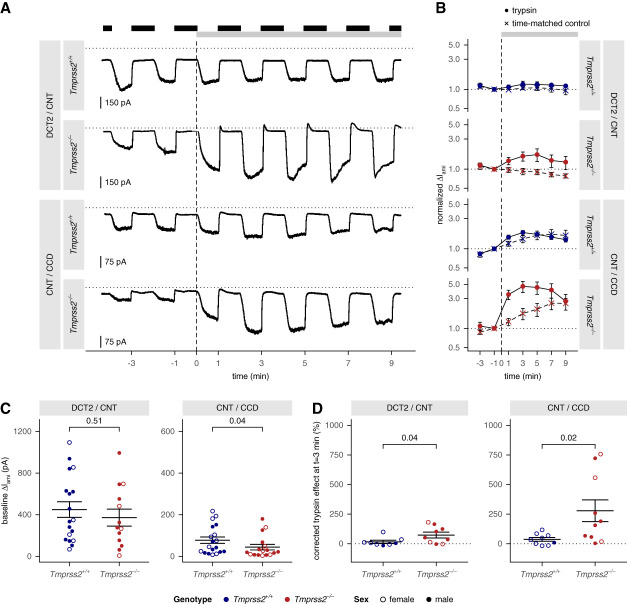
**Whole-cell current recordings in microdissected tubules revealed impaired proteolytic ENaC activation in *Tmprss2***^***−/−***^ **mice.** (A) Representative whole-cell current traces are shown from DCT2/CNT and CNT/CCD isolated from *Tmprss2*^+/+^ and *Tmprss2*^−/−^ mice, as indicated. Presence of amiloride (4 *µ*M) and trypsin (20 *µ*g/ml) in the bath solution is indicated by black and gray bars, respectively. Unless trypsin was added, all bath solutions contained 2 *μ*g/ml SBTI to reduce the risk of a contamination with trypsin. A dotted line indicates zero current level. Timepoint of trypsin application or of mock solution exchange in time-matched control experiments is referred to as 0 minute and marked with a dashed vertical line. (B) Summary of normalized amiloride-sensitive current values (ΔI_ami_) from similar experiments as shown in (A) with trypsin application (filled circles) and from time-matched control recordings shown in Supplemental Figure 23 (crosses) displayed on a logarithmic scale. For each cycle of amiloride washout and reapplication, ΔI_ami_ was calculated by subtracting the current value measured immediately before amiloride application from that reached in the presence of amiloride. ΔI_ami_ values determined at different time points in each individual recording were normalized to the ΔI_ami_ obtained in the second cycle of the same experiment (at *t*=−1), that is, from the amiloride application just before trypsin application or mock solution exchange. Absolute ΔI_ami_ values are shown in Supplemental Figure 23B. It is noteworthy that the slow time course of spontaneous current runup observed in time-matched control recordings in CNT/CCD was clearly different from the rapid stimulatory response to trypsin, which usually reached a maximum within 3 to 5 minutes followed by a gradual current decline. The latter probably resulted from ENaC degradation during prolonged trypsin exposure. Mean±SEM are shown with symbols and error bars. *P* values from the Kruskal–Wallis test of log-transformed values comparing trypsin application and time-matched control: 0.1 (first), <0.001 (second), 0.6 (third), 0.02 (fourth). (C) Summary of baseline ΔI_ami_ values obtained in the second cycle of amiloride washout/reapplication (*t*=−1) from the same experiments shown in (A) and (B) and in Supplemental Figure 23. Mean±SEM and individual data points are shown. Sex is indicated with open (female) and closed (male) symbols. One-sided Wilcoxon signed-rank test with Bonferroni correction for multiple testing. (D) Summary of corrected normalized effects of trypsin on ΔI_ami_ 3 minutes after its application from experiments shown in (A) and (B). To account for spontaneous changes in ΔI_ami_, each normalized ΔI_ami_ value at *t*=3 minutes obtained from trypsin-treated cells was corrected by subtracting the average normalized ΔI_ami_ at *t*=3 minutes from corresponding time-matched control recordings (see Supplemental Figure 23A). Mean±SEM and individual data points are shown. One-sided Wilcoxon signed-rank test of log-transformed values with Bonferroni correction for multiple testing. CCD, cortical collecting duct; CNT, connecting tubule; DCT2, distal convoluted tubule; SBTI, soybean trypsin inhibitor.

To explore the relevance of TMPRSS2 for ENaC function in native renal tubules, we performed whole-cell patch-clamp recordings in DCT2/CNT and CNT/CCD from *Tmprss2*^−/−^ and wild-type control mice (Figure 6 and Supplemental Figure 23). Repeated amiloride applications were used to monitor ENaC-mediated currents (ΔI_ami_). To reveal the presence of incompletely cleaved channels at the cell surface, the current response to apical trypsin application was investigated using a previously established protocol.^56^

In both genotypes, average initial ΔI_ami_ values in CNT/CCD were significantly lower than corresponding values in DCT2/CNT (Figure 6, A and C). In DCT2/CNT, we observed a trend toward reduced baseline ΔI_ami_ in *Tmprss2*^−/−^ mice (372±81 pA) compared with control mice (449±75 pA; Figure 6C). Apical application of trypsin to DCT2/CNT from *Tmprss2*^*−/−*^ mice increased ΔI_ami_ on average by approximately 75% within about 3 minutes (Figure 6, B and D). By contrast, no trypsin response was observed in DCT2/CNT from wild-type animals (Figure 6, A, B, and D, and Supplemental Figure 23B). In CNT/CCD, baseline ENaC currents were significantly lower in *Tmprss2*^−/−^ mice (45±13 pA) than those in wild-type mice (78±15 pA; Figure 6C). The latter currents were slightly stimulated by application of trypsin (approximately 40%). Importantly, in CNT/CCD from *Tmprss2*^−/−^ mice, the stimulatory effect of trypsin was much larger with an average increase of ENaC currents by approximately 280% within 3 minutes (Figure 6, B and D).

## Discussion

Key findings of this study were the following: (*1*) coexpression of murine TMPRSS2 proteolytically activated murine ENaC by cleaving its *γ*-subunit consistent with our previous findings with the human orthologs^28^; (*2*) TMPRSS2 deficiency reduced baseline ENaC activity in mCCD_cl1_ cells and the stimulatory effect of aldosterone because of incomplete *γ*-ENaC cleavage; (*3*) in *Tmprss2*^−/−^ mice, renal ENaC cleavage was compromised, and animals required a much larger increase in plasma aldosterone to reduce renal sodium excretion adequately in response to dietary sodium restriction, in particular when combined with increased potassium intake; and (*4*) renal ENaC whole-cell currents could be stimulated by trypsin to a larger extent in *Tmprss2*^−/−^ mice than in wild-type mice, particularly in the CNT/CCD. This indicated that TMPRSS2 deficiency reduced average open probability of ENaC probably because of impaired proteolytic channel activation. Collectively, our results support the conclusion that TMPRSS2 is a functionally important protease coexpressed with ENaC in distal tubular epithelial cells.

The additional stimulatory effect of chymotrypsin on baseline *I*_SC_ provided functional evidence for incomplete proteolytic ENaC activation in TMPRSS2-deficient mCCD_cl1_ cells. The finding that the fraction of fully cleaved *γ*-ENaC at the cell surface of TMPRSS2-deficient cells was lower than in control cells further confirmed the concept that TMPRSS2 is essential for complete *γ*-ENaC cleavage. Importantly, the stimulatory effect of aldosterone on *I*_SC_ was reduced in TMPRSS2-deficient mCCD_cl1_ cells. ENaC regulation by aldosterone is highly complex^3,9^ and involves the stimulation of channel trafficking to the apical membrane and enhanced proteolytic ENaC cleavage.^47,52–55^ Our findings indicated that TMPRSS2 deficiency did not impede the stimulatory effect of aldosterone on channel insertion into the apical membrane, but prevented full proteolytic activation of the newly inserted channels. This was evidenced by the finding that in TMPRSS2-ko mCCD_cl1_ cells, the stimulatory effect of chymotrypsin was enhanced after a 2-hour exposure to aldosterone. RNA-seq analysis did not reveal a prominent regulatory effect of aldosterone on the transcriptional expression of TMPRSS2 or any other serine protease detected, in line with previous research.^51,57,58^ Moreover, aldosterone treatment did not enhance TMPRSS2 expression or proteolytic activity detected in the apical medium from mCCD_cl1_ cells. To conclude, increased TMPRSS2 expression or function was not required to achieve full proteolytic ENaC activation after aldosterone stimulation. Thus, in mCCD_cl1_ cells, proteolytic activity of constitutively expressed TMPRSS2 seems to be sufficient to process all channels trafficking to the cell surface under baseline and aldosterone-stimulated conditions. This supports the concept that ENaC cleavage depends on the regulation of channel trafficking.^53,54^ However, this does not rule out the possibility that long-term stimulation of ENaC activity *in vivo* also involves upregulation of protease activity.

Importantly, we demonstrated in microdissected tubules that ENaC could be stimulated by trypsin to a larger extent in *Tmprss2*^−/−^ mice than in wild-type mice. Indeed, the approximately 280% stimulation of ENaC currents by trypsin in CNT/CCD of *Tmprss2*^−/−^ mice was much more pronounced than in wild-type mice, where trypsin had only a modest (approximately 40%) stimulatory effect on ENaC, consistent with previously reported findings.^55,56^ Interestingly, in DCT2/CNT of wild-type mice, trypsin had no apparent effect but significantly increased ENaC currents in DCT2/CNT of *Tmprss2*^−/−^ mice by approximately 75%. The different responsiveness of ENaC currents to trypsin in DCT2/CNT versus CNT/CCD in the presence and absence of TMPRSS2 suggests a site-specific role of TMPRSS2 in proteolytic ENaC processing. This implies that in addition to TMPRSS2, other proteases contribute to proteolytic ENaC activation, possibly in a site-specific manner. Site-specific differences in renal ENaC regulation are increasingly being recognized. In this context, it is noteworthy that in *Tmprss2*^−/−^ mice, like in wild-type mice, baseline ENaC currents in DCT2/CNT were significantly larger than those in CNT/CCD. This is in good agreement with the emerging concept that, unlike in CNT/CCD, baseline ENaC currents in DCT2/CNT are aldosterone-independent, albeit not independent of the mineralocorticoid receptor.^6–11,59^ In conclusion, our patch-clamp studies in microdissected tubules indicated that in TMPRSS2-deficient mice, proteolytic ENaC activation at the apical surface of tubular cells was incomplete mainly in CNT/CCD but also in DCT2/CNT.

In kidney tissue from *Tmprss2*^−/−^ mice maintained on standard diet, the fraction of partially cleaved *γ*-ENaC was increased compared with wild-type controls. It is tempting to speculate that a pool of incompletely cleaved *γ*-ENaC builds up to compensate for the insufficient generation of fully cleaved *γ*-ENaC because of TMPRSS2 deficiency. Indeed, TMPRSS2-deficient mice had no overt phenotype and adequately reduced their renal sodium excretion when challenged with a low-sodium diet. Moreover, under baseline conditions as well as under sodium restriction, their natriuretic response to acute administration of amiloride was similar to that of wildtype mice. Thus, overall ENaC activity in TMPRSS2-deficient mice was similar to that in wild-type mice. This indicated that animals were able to compensate for impaired *γ*-ENaC cleavage caused by TMPRSS2 deficiency.

Compensation for TMPRSS2 deficiency was probably achieved by an increased stimulation of the renin-angiotensin-aldosterone axis. This was evidenced by the significantly higher aldosterone levels reached in TMPRSS2-deficient mice compared with control mice when animals were challenged with a sodium-deficient diet, in particular in combination with a high potassium intake. The latter combination is a particularly strong stimulus for aldosterone secretion and ENaC activation. In this context, it should be noted that a local increase in basolateral potassium concentration may also directly stimulate renal ENaC activity.^60^ Moreover, angiotensin II can stimulate ENaC by aldosterone-independent mechanisms in particular in DCT2/CNT,^59^ and additional factors independent of *γ*-ENaC cleavage may modulate ENaC open probability.^61,62^

Interestingly, Western blot analysis of kidney tissue from sodium-restricted mice did not reveal differences in overall ENaC expression and *γ*-ENaC cleavage pattern between wild-type versus TMPRSS2-deficient mice. However, with our experimental approach, we cannot distinguish between ENaC in the cytosol and at the apical membrane. Moreover, unlike our patch-clamp experiments in microdissected tubules, our Western blot analysis could not distinguish between ENaC expression in DCT2/CNT versus CNT/CCD. Thus, we may have missed subtle differences between wild-type and *Tmprss2*^−/−^ mice regarding the expression level and cleavage state of *γ*-ENaC, in particular at the apical cell surface in CNT/CCD. Moreover, additional endogenous proteases may contribute to ENaC activation in sodium-restricted mice and may at least in part compensate for the loss of TMPRSS2 activity, in particular in the DCT2/CNT.

During preparation of this article, a study was published on TMPRSS2-ko mCCD_cl1_ cell lines generated by clonal selection.^34^ TMPRSS2 deficiency in these cell clones was associated with a strong downregulation of *α*-ENaC mRNA expression that essentially abrogated ENaC currents.^34^ By contrast, in our study, we generated polyclonal TMPRSS2-ko cells to avoid any clonal off-target effects. In our TMPRSS2-ko mCCD_cl1_ cells, we could reliably detect amiloride-sensitive *I*_SC_ and observed no transcriptional downregulation of *α*-ENaC. Thus, the different methods of cell line generation might explain the different results obtained in the two studies. Importantly, our Western blot analysis (Figure 4 and Supplemental Figures 14, 15, 21, and 22) and immunostaining experiments in mouse kidney (Figures 3 and 5A), as well as the observation that the natriuretic response to amiloride was similar in *Tmprss2*^+/+^ and *Tmprss2*^−/−^ mice (Supplemental Figure 16), argue against a substantial downregulation of ENaC expression by TMPRSS2-ko *in vivo*.

Increased ENaC activity is likely to contribute to the pathophysiology of essential hypertension, in particular in a subset of patients with salt-sensitive hypertension.^63^ TMPRSS2 may emerge as novel pharmacological target to reduce ENaC activity in the context of hypertension. Indeed, previous studies have demonstrated a BP-lowering effect of the protease inhibitor camostat mesylate in Dahl salt-sensitive rats.^64,65^ This was attributed to an attenuation of proteolytic ENaC activation.

In summary, our data show that TMPRSS2 contributes to proteolytic ENaC activation in the kidney, particularly in CNT/CCD where ENaC activity is aldosterone-dependent. In the future, the development of specific TMPRSS2 inhibitors^66,67^ may open new therapeutic perspectives to limit proteolytic ENaC activation in the kidney in disease states with inappropriately high ENaC activity.

## Supplementary Material

## Data Availability

Data related to transcriptomic, proteomic, or metabolomic data. Raw Data/Source Data. Gene Expression Omnibus (GEO). GEO accession GSE274304: https://www.ncbi.nlm.nih.gov/geo/query/acc.cgi?acc=GSE274304. GEO accession GSE274305: https://www.ncbi.nlm.nih.gov/geo/query/acc.cgi?acc=GSE274305. GEO accession GSE274306: https://www.ncbi.nlm.nih.gov/geo/query/acc.cgi?acc=GSE274306.

## Supplemental Material

Supplemental Methods

Supplemental Figure 1. Validation of anti-TMPRSS2 antibody in *Xenopus laevis* oocytes.

Supplemental Figure 2. Murine ENaC was proteolytically activated by coexpression of murine TMPRSS2 in *Xenopus laevis* oocytes with a bell-shaped dependence of amiloride-sensitive ENaC currents on the injected amount of TMPRSS2 cRNA.

Supplemental Figure 3. Western blot detection of TMPRSS2 in mCCD_cl1_ cells.

Supplemental Figure 4. TMPRSS2 knockout in mCCD_cl1_ cells did not affect normal epithelial monolayer formation in culture.

Supplemental Figure 5. TMPRSS2 was present in the apical medium of mCCD_cl1_ cells.

Supplemental Figure 6. Chymotrypsin failed to stimulate ENaC in the presence of amiloride in TMPRSS2-knockout and control mCCD_cl1_ cells.

Supplemental Figure 7. Apically applied aprotinin reduced *I*_SC_ in control but not in TMPRSS2-ko mCCD_cl1_ cells.

Supplemental Figure 8. Western blot detection of *γ*-ENaC cleavage fragments in mCCD_cl1_ cells.

Supplemental Figure 9. RNA-sequencing analysis of mCCD_cl1_ cells treated over 2 or 24 hours with aldosterone.

Supplemental Figure 10. Control *I*_SC_ recordings in mCCD_cl1_ cells used for RNA-sequencing analysis.

Supplemental Figure 11. Aldosterone did not increase TMPRSS2 abundance at the cell surface or in apical medium of control mCCD_cl1_ cells.

Supplemental Figure 12. RNA sequencing analysis of control and TMPRSS2-ko mCCD_cl1_ cells.

Supplemental Figure 13. Confirmation of TMPRSS2 knockout in *Tmprss2*^−/−^ kidneys.

Supplemental Figure 14. Original Western blots of *γ*-ENaC in mouse kidney cortex.

Supplemental Figure 15. In *Tmprss2*^−/−^ mice, proteolytic processing of renal *α*-ENaC was not reduced but rather increased in comparison with *Tmprss2*^+/+^ mice.

Supplemental Figure 16. Natriuretic response to amiloride is preserved in *Tmprss2*^−/−^ mice.

Supplemental Figure 17. Plasma Na^+^ and K^+^ concentrations, urinary K^+^ excretion, and fecal Na^+^ excretion in response to dietary sodium restriction were not different in *Tmprss2*^−/−^ compared with wild-type mice.

Supplemental Figure 18. Like *Tmprss2*^*+/+*^ control mice, *Tmprss2*^−/−^ mice maintained body weight under low-sodium diet with similar food intake but higher water intake and urinary output.

Supplemental Figure 19. In response to low-sodium diet in combination with increased potassium intake, *Tmprss2*^−/−^ mice maintained sodium and potassium balance.

Supplemental Figure 20. Under low-sodium diet in combination with increased potassium intake, body weight, food intake, water intake, and urine output were similar in *Tmprss2*^*+/+*^ and *Tmprss2*^−/−^ mice.

Supplemental Figure 21. Analysis of proteolytic processing of renal *γ*-ENaC under low-salt diet.

Supplemental Figure 22. Analysis of proteolytic processing of renal *α*-ENaC under low-salt diet.

Supplemental Figure 23. Representative time-matched control recordings from microdissected tubules and summary of ΔI_ami_ time courses in absolute values from individual experiments.

Supplemental Table 1. Significantly upregulated gene transcripts in mCCD_cl1_ cells treated for 2 hours with 3 nM aldosterone.

Supplemental Table 2. Significantly upregulated and downregulated transcripts in mCCD_cl1_ cells treated for 24 hours with 3 nM aldosterone.

Supplemental Table 3. Differentially expressed transcripts in TMRPSS2-ko versus control mCCD_cl1_ cells.
