# Supplementary material for: Transmembrane Serine Protease 2 and Proteolytic Activation of the Epithelial Sodium Channel in Mouse Kidney
Source: J Am Soc Nephrol. 2024 Oct 23;36(3):420–34. doi: 10.1681/ASN.0000000521 (PMC11888964; doi:10.1681/ASN.0000000521)
Supplement: Supplementary file 2 [file jasn-36-420-s002.pdf]

Transmembrane serine protease 2 and proteolytic activation of the epithelial sodium channel in mouse kidney

Supplemental Material Table of Contents

Supplemental Methods
Supplemental Figure 1: Validation of anti-TMPRSS2 antibody in *X. laevis* oocytes.
Supplemental Figure ~~2: Western blot detection of TMPRSS2 in $mCCD_{cl1}$ cells~~
Supplemental Figure ~~3: Murine ENaC was proteolytically activated by coexpression of murine TMPRSS2 in *X. laevis* oocytes with a bell-shaped dependence of amiloride-sensitive ENaCcurrents on the injected amount of TMPRSS2 cRNA~~
Supplemental Figure 4: TMPRSS2 knockout in mCCD~~cl1~~ cells did not affect normal epithelial monolayer formation in culture
Supplemental Figure 5: TMPRSS2 was present in the apical medium of $mCCD_{cl1}$ cells
Supplemental Figure 6: Chymotrypsin failed to stimulate ENaC in the presence of amiloride in TMPRSS2-knockout and control $mCCD_{cl1}$ cells
Supplemental Figure 7: Apically applied aprotinin reduced *I*~~SC~~ in control but not in TMPRSS2-ko $mCCD_{cl1}$ cells.
Supplemental Figure 8: Western blot detection of γ-ENaC cleavage fragments in $mCCD_{cl1}$ cells
Supplemental Figure 9: RNA-sequencing analysis of mCCD~~cl1~~ cells treated over 2 h or 24 h with aldosterone
Supplemental Figure 10: Control *I*~~SC~~ recordings in mCCD~~cl1~~ cells used for RNA-sequencing analysis.
Supplemental Figure 11: Aldosterone did not increase TMPRSS2 abundance at the cell surface or in apical medium of control $mCCD_{cl1}$ cells.
Supplemental Figure 12: RNA sequencing analysis of ~~C~~ontrol and TMPRSS2-ko mCCD~~cl1~~ cells
Supplemental Figure 13: Confirmation of TMPRSS2 knockout in *Tmprss2*~~−/−~~ kidneys
Supplemental Figure 14: Original western blots of γ-ENaC in mouse kidney cortex
Supplemental Figure 15: In *Tmprss2*~~−/−~~ mice, proteolytic processing of renal α-ENaC was not reduced but rather increased in comparison with *Tmprss2*~~+/+~~ mice
Supplemental Figure 16: Natriuretic response to amiloride is preserved in *Tmprss2*~~−/−~~ mice
Supplemental Figure 17: Plasma Na^+ and K^+ concentrations, urinary K^+ excretion and fecal Na^+ excretion in response to dietary sodium restriction were not different in *Tmprss2*~~−/−~~ compared to wildtype mice
Supplemental Figure 18: Like *Tmprss2*~~+/+~~ control mice, *Tmprss2*~~−/−~~ mice maintained body weight under low sodium diet with similar food intake but higher water intake and urinary output
Supplemental Figure 19: In response to low sodium diet in combination with increased potassium intake, *Tmprss2*~~−/−~~ mice maintained sodium and potassium balance

Supplemental Figure 20: Under low sodium diet in combination with increased potassium intake body weight, food intake, water intake and urine output were similar in *Tmprss2*+/+ and *Tmprss2*−/− mice
Supplemental Figure 21: Analysis of proteolytic processing of renal γ-ENaC under low salt diet
Supplemental Figure 22: Analysis of proteolytic processing of renal α-ENaC under low salt diet
Supplemental Figure 23: Representative time-matched control recordings from microdissected tubules and summary of ΔI_{ami} time-courses in absolute values from individual experiments
Supplemental Table 1. Significantly upregulated gene transcripts in $mCCD_{cl1}$ cells treated for 2 h with 3 nM aldosterone
Supplemental Table 2. Significantly up- and downregulated transcripts in $mCCD_{cl1}$ cells treated for 24 h with 3 nM aldosterone
Supplemental Table 3. Differentially expressed transcripts in TMRPSS2-ko *vs.* control $mCCD_{cl1}$ cells

Supplemental Methods

Two-electrode voltage-clamp experiments in Xenopus laevis oocytes

Full-length complementary DNAs (cDNAs) encoding murine α-, β-, and γ-ENaC were kindly provided by Prof. Marcus A. Mall (current affiliation: Charité – Universitätsmedizin Berlin, Berlin, Germany). A full-length cDNA encoding murine TMPRSS2 was obtained from GenScript Biotech. cDNAs were subcloned into the pTLN vector for heterologous expression in *Xenopus laevis* oocytes [Lorenz C, Pusch M, Jentsch TJ. Heteromultimeric CLC chloride channels with novel properties. *Proc Natl Acad Sci U S A*. 1996; 93(23):13362-13366. doi:10.1073/pnas.93.23.13362]. Plasmids were linearized and used as templates for cRNA synthesis using SP6 RNA polymerase (mMessage mMachine, Ambion). QuickChange Lightning site-directed mutagenesis kit (Agilent Technologies) was used to attach a 3' HA-tag (YPYDVPDYA) to TMPRSS2. Sequences were routinely confirmed by sequence analysis (LGC Genomics).

Ovarian lobes were excised by partial ovariectomy under anesthesia with Tricain 0.2%, in accordance with the principles of German legislation, with approval by the animal welfare officer for the University of Erlangen-Nürnberg (FAU), and under the governance of the state veterinary health inspectorate. Oocytes were isolated from ovarian lobes using a type-2 collagenase from *Clostridium histolyticum* (Sigma-Aldrich). Defolliculated stage V-VI oocytes were injected with 0.1 ng of cRNA per ENaC subunit (α, β, and γ) without or with cRNA encoding for 3'HA-tagged murine TMPRSS2 (0.2 ng if not stated otherwise) per oocyte. After cRNA injection, oocytes were kept in a low sodium ND9 solution (composition in millimolar: 9 NaCl, 2 KCl, 87 N-methyl-D-glutamine-Cl, 1.8 $CaCl_2$, 1 $MgCl_2$, 5 HEPES, and pH 7.4 adjusted with Tris) supplemented with 100 units/ml sodium penicillin and 100 µg/ml streptomycin sulfate. Two-electrode voltage-clamp (TEVC) measurements were performed 48 h after cRNA injection. ENaC-mediated whole-cell currents (ΔI_{ami}) were determined by washing out amiloride (2 µM; Sigma-Aldrich) with amiloride-free bath solution and subtracting the averaged whole-cell currents measured in the presence of amiloride at the beginning and the end of each recording from the corresponding whole-cell currents recorded in its absence. ND96 was used as a standard bath solution (composition in millimolar: 96 NaCl, 2 KCl, 1.8 $CaCl_2$, 1 $MgCl_2$, 5 HEPES; pH 7.4 adjusted with Tris). Chymotrypsin (α-chymotrypsin type II; 2 µg/ml; Sigma-Aldrich) was added to the bath solution to assess proteolytic ENaC activation.

TMPRSS2 knockout in $mCCD_{cl1}$ cells and Ussing chamber measurements

The sgRNA sequence targeting exon 6 of *Tmprss2* (AGAGCAGCATGTAAAGACAT) was designed using GPP sgRNA Designer (https://portals.broadinstitute.org/gpp/public/analysis-tools/sgrna-design) and cloned into the pLenti-CRISPR-V2 vector as described previously[41,42]. The following sgRNA sequence targeting firefly luciferase, which is not expressed in murine cells, was used to generate nontargeting control $mCCD_{cl1}$ cells: TACAAACGCTCTCATCGACA. pLenti-CRISPR-V2 was a gift from Feng Zhang (Addgene plasmid #52961; Research Resource Identifier: Addgene 52961). To generate CRISPR/Cas9-V2-based lentiviral particles, HEK 293T cells were transfected with vesicular stomatitis virus glycoprotein expression plasmid (pVSV-G), the HIV

gag/pol packaging plasmid pCMVΔR8.9, and lentiviral vector pLenti-CRISPR-V2 encoding corresponding sgRNAs at a mass ratio of 1:2:2 using calcium phosphate. Cell-culture supernatants were collected at 48 hours postinfection, passed through 0.45 µm pore size filters, concentrated by centrifugation through size-exclusion filters (Amicon, Millipore), and stored at −80 °C. Three days postinfection, transduced $mCCD_{cl1}$ cells were selected using 2.5 µg/ml puromycin-containing medium.

Wildtype (passages 29-36), non-targeting control (passages 33-40), and TMPRSS2-knockout $mCCD_{cl1}$ cells (passages 33-40) were cultured in parallel, essentially as described previously[44]. Cells were maintained in a 5% CO_2 atmosphere at 37 °C in phenol-red free DMEM/F12 medium supplemented with 2% fetal bovine serum, 1 nM triiodothyronine, 60 nM sodium selenite, 5 µg/ml apotransferrin, 10 ng/ml epidermal growth factor, 5 µg/ml insulin, 50 nM dexamethasone, 100 units/ml penicillin, 100 µg/ml streptomycin. For experiments, cells were seeded on permeable supports (Millicell PCF membrane inserts; Merck-Millipore) in a standard culture medium. After five days, cells were fed with modified medium lacking apotransferrin, epidermal growth factor, and fetal bovine serum. 24 h before experiments, dexamethasone was removed from the supplemented medium, and medium was exchanged for the last time. 9-11 days after seeding, monolayers were transferred into Ussing chambers to measure the equivalent short circuit current (I_{SC}) and transepithelial electrical resistance (TEER) as previously described[43,44]. A 0.9% NaCl solution of chymotrypsin (2 mg/ml), aprotinin (3 mg/ml) or amiloride (1 mM) was added directly to the apical bath solution to achieve the final concentration of 20 µg/ml, 30 µg/ml or 10 µM, respectively. A 1 mM aldosterone (Sigma-Aldrich) stock solution was prepared by dissolving it in ethanol. This stock solution was further diluted with 0.9% NaCl to obtain a 300 nM aldosterone solution. An aliquote of the latter solution was added to both apical and basolateral compartments to achieve a final aldosterone concentration of 3 nM. The final ethanol concentration was negligible (0.0003%). Therefore, 0.9% NaCl was used as a vehicle control for aldosterone applications.

Determination of trypsin-like proteolytic activity

Trypsin-like proteolytic activity in apical medium of $mCCD_{cl1}$ cells was assessed using the fluorogenic substrate Boc-Gln-Ala-Arg-AMC (Boc-QAR-AMC; Boc: t-Butyloxycarbonyl; AMC: 7-Amino-4-methyl-coumarin; R&D systems, Abingdon, UK). Measurements were performed 9-11 days after cell seeding and 12 h (Figure 1) or 3 h (Figure 2) after the last medium exchange. 100 µl medium samples were taken from the apical compartment of individual permeable supports, supplemented with 10 µM fluorogenic substrate, and transferred to a 96-well plate. The fluorescence signal (360 nm excitation, 465 nm emission wavelength) from substrate hydrolysis was recorded in 10 min intervals over 190 min using a TECAN GENios plate reader (Tecan). For experiments shown in Figure 2, apical medium was collected before cells were harvested from permeable supports and lyzed to estimate the total protein concentration using BCA assay. This ensured that cell density was roughly similar in control and TMPRSS2-knockout $mCCD_{cl1}$ cells.

RNA-sequencing

For experiments shown in Supplemental Figure 9, total RNA was isolated from wildtype $mCCD_{cl1}$ cells treated with 3 nM aldosterone or vehicle (0.9% NaCl) for either 2 h or 24 h. I_{SC}

was continuously recorded in modified Ussing chambers for 2 h aldosterone applications (Supplemental Figure 10A-C) or was determined in spot measurements using a commercially available epithelial volt-ohm meter (EVOM) and a set of two sticks "STX" electrodes (World Precision Instruments) in experiments with 24 h aldosterone applications (Supplemental Figure 10D-F). To minimize Na^+-feedback inhibition, apical amiloride (2 μM) was present during the 24 h aldosterone exposure and removed shortly before RNA-isolation to determine the amiloride-sensitive I_{SC} component. For RNA-seq experiments shown in Supplemental Figure 12, total RNA was isolated from non-targeting control and TMPRSS2-deficient $mCCD_{cl1}$ cells following electrophysiological experiments (Figure 1).

RNA was isolated from cells harvested from each individual permeable support using the NucleoSpin RNA kit (Machery-Nagel), resulting in a total of 6 (Supplemental Figure 9A, C, E and Supplemental Figure 12) or 7 (Supplemental Figure 9B, D, F) RNA samples per experimental condition. RNA sequencing was performed at the Next Generation Sequencing Core Unit (Institute of Human Genetics, Faculty of Medicine, FAU Erlangen-Nürnberg). The mRNA libraries were prepared using the Illumina Truseq Stranded mRNA kit and sequenced on the Illumina Hiseq 2500 and Illumina Novaseq 6000 platforms. After demultiplexing, quality control was performed with FastQC v0.11.8. As there were no systemic QC issues and the overall read quality was very high, reads were mapped to the *Mus musculus* reference genome (Ensembl GRCm38.102) using STAR v 2.7 after quality trimming with cutadapt v1.15. Raw counts were obtained using subread featureCounts v2.0.1 and the Ensembl GRCm38.102 annotation, subsequent differential expression analysis was performed using DESeq2 v1.3. Genes with a false discovery rate ≤ 0.05 and a fold-change of at least 2 were considered differentially expressed. To obtain Transcripts per Million (TPM), reads were quantified using Salmon v1.10.3 and the combined Ensembl GRCm38.102 cdna and ncrna references.

Mouse studies

An established TMPRSS2-knockout mouse model (global constitutive TMPRSS2-knockout; background: C57BL/6J), in which exons 10 to 13 encoding the catalytic domain of TMPRSS2 were deleted[46], was obtained from Jackson Laboratories (B6.129-*Tmprss2*^{tm1Psn}/ J, JAX stock #026196). Genotyping was performed by DNA-extraction from mouse ear biopsies and subsequent PCR analysis as described in The Jackson Laboratory Genotyping Protocol Database (protocol 28215). TMPRSS2-wildtype littermate mice served as controls for all experiments.

For experiments shown in Figures 3, 4, 6, Supplemental Figures 13-15, and Supplemental Figure 23, animals were bred and maintained in the animal facility of Friedrich-Alexander-Universität Erlangen-Nürnberg, and all animal procedures were performed in accordance with the principles of German legislation, with approval by the animal welfare officer for the University of Erlangen-Nürnberg and under the governance of the state veterinary health inspectorate (approval numbers: TS-11/2017, TS-10/2022). Mice of both sexes were used at ages of 10-48 weeks. Mice were kept on a 12:12-h light-dark cycle with tap water *ad libitum*. Mice received a standard diet (Na^+ content: 3.2 g/kg and K^+: 10.2 g/ kg, Cat. No. 1310, Spezialfutter, Lage, Germany). Animals were anesthetized with ketamine and xylazine overdosage (300 mg/kg ketamine and 60 mg/kg xylazine) or isoflurane (4%) and euthanized by cervical dislocation. Subsequently kidneys were harvested for further analysis.

For experiments shown in Figure 5 and Supplemental Figures 16-22 animals were bred and maintained at the animal facility of the University of Tübingen and experiments were conducted according to German law and with approval by local authorities (Regierungspräsidium Tübingen, approval number M 22/21G). Experiments were performed on 3-month-old mice. Mice received a standard chow (ssniff, V1534, Soest, Germany) and were maintained on a 12:12 hour light-dark cycle with *ad libitum* access to tap water. Acute responses to amiloride 10 µg/g BW (body weight), or vehicle were studied by bolus administration i.p. 5 µl/g BW and subsequent collection of urine for 6 h. To investigate the effect of a low sodium diet with and without high potassium, mice were studied in metabolic cages. Mice were acclimated in the metabolic cages for 3 and 6 hours prior to the start of the study, followed by an acclimation and a control day with a control diet (C1000, sodium and potassium content 110 $\mu mol \cdot g^{-1}$ and 178 $\mu mol \cdot g^{-1}$, respectively, Altromin, Lage, Germany). The mice were then treated with the low sodium diet (C1036, sodium and potassium content 10 $\mu mol \cdot g^{-1}$ and 178 $\mu mol \cdot g^{-1}$) with or without high potassium (1 tablet Kalinor^{TM} dissolved in 400 ml drinking water, final concentration 100 mM) for 4 consecutive days. To reduce stress, the mice were provided with a shelter. The amount of food, water and urine was measured daily and the body weight of the mice was recorded. Urine was frozen at −20°C until further use. Blood was collected under isoflurane anesthesia by puncture of the retrobulbar plexus, collected in heparinized vials, measured by blood gas analysis or centrifuged, and the plasma stored at −20°C. Kidneys were removed after cervical dislocation and complete bleeding of the animals. The kidneys were shock frozen in liquid nitrogen and stored at −80°C. Urinary creatinine was measured with a colorimetric Jaffé assay (Labor+Technik, Berlin, Germany). Plasma sodium and potassium were measured using an IL GEM® Premier 3000 blood gas analyzer (Instrumentation Laboratory, Munich, Germany). Urinary sodium and potassium concentrations were measured *via* flame photometry (Eppendorf EFUX 5057, Hamburg, Germany).

Preparation of Renal Tubules and Electrophysiology

After cervical dislocation under anesthesia with isoflurane (4%), thorax and abdomen were opened. Vena cava inferior was cut open and the circulatory system was perfused via the left ventricle with Leibowitz medium (Thermo Fisher Scientific, Schwerte, Germany) containing 350 U/ml collagenase (Biochrom CLS IV). Kidneys were harvested, coronary slices were prepared and incubated in Leibowitz medium containing collagenase at 37 °C for 20-30 min. After incubation with collagenase, slices were kept in ice-cold Leibowitz medium. Microdissected fragments of the distal nephron were placed on small pieces of glass cover slips covered with Cell-Tak (Corning, Kaiserslautern, Germany) and were transferred to a flow chamber positioned on the microscope stage of the patch-clamp setup. To gain access to the apical membrane, tubules were cut open with a broken glass pipette attached to a micromanipulator. Patch-clamp experiments were performed in two distinct regions of the distal nephron 1) DCT2 and initial CNT (DCT2/CNT), and 2) late CNT and initial CCD (CNT/CCD), which were identified according to morphological criteria as previously described[6].

Patch-clamp experiments were performed essentially as previously described[7,10,53]. An EPC-9 patch-clamp amplifier controlled with PatchMaster software (HEKA Elektronik, Lambrecht, Germany) was used. Pipettes were made from borosilicate glass (Hilgenberg,

Malsfeld, Germany) with a resistance of about 4-6 MΩ. To minimize noise due to currents through Cl^- channels, the Cl^- concentrations were kept low in both extracellular bath and intracellular pipette solutions. The pipette solution contained (composition in millimolar): 5 Na gluconate, 85 K gluconate, 40 CsOH, 20 Tetraethylammonium-OH, 2 ATP Mg salt, 2 $MgCl_2$, 2 EGTA Na, 10 HEPES and was titrated to pH 7.2 with gluconic acid. Bath solution had the following composition (in millimolar): 140 Na gluconate, 5 K gluconate, 2 $CaCl_2$, 1 $MgCl_2$, 10 Ba acetate, 10 HEPES, titrated with TRIS to pH 7.4. Amiloride was used in a concentration of 4 µM. Unless trypsin was added, all bath solutions contained 2 µg/ml soybean trypsin inhibitor (SBTI) to reduce the risk of a contamination with trypsin. For the continuous recording of inward Na^+ currents a holding potential (V_{hold}) of −60 mV was used. Experiments were performed at 37 °C by using pre-heated solutions and controlling the temperature of the perfusion chamber. Whole cell currents were recorded with a sampling rate of 4000 Hz and antialiasing Bessel filtering at 800 Hz. Subsequently, recordings were digitally re-filtered for the analysis at frequencies of 100-250 Hz depending on the signal quality. Electrophysiological recordings were analysed using Nest-o-patch (https://sourceforge.net/projects/nestopatch/).

Immunoblotting

To separate cell surface proteins from intracellular proteins in oocytes, an established biotinylation approach was used[28].

For analysis in apical medium of $mCCD_{cl1}$ cells, the medium was collected from the apical compartment of $mCCD_{cl1}$ 9-11 days after cell seeding and 12 h after the last medium exchange for experiments shown in Supplemental Figure 5 or 3 h after the last medium exchange for experiments shown in Supplemental Figure 11B. Proteins were precipitated by adding four times the sample volume of acetone and incubating for 1 h at −20 °C. After 10 min centrifugation at 15,000*g*, supernatant was discarded and dried pellet was resuspended in a buffer (composition in millimolar: 50 Hepes, 150 NaCl, 10% glycerol, 1% Triton X-100) for a western blot analysis.

Whole-cell lysates from $mCCD_{cl1}$ cells were obtained by scraping cells from their permeable supports following Ussing chamber measurements and by transferring them into a lysis buffer (composition: 50 mM Hepes, 150 mM NaCl, 10% glycerol, 1% Triton X-100) supplemented with protease inhibitor mixture (cOmplete EDTA-free protease inhibitor mixture tablets; Roche diagnostics). Samples were loaded after sonication and 10 min centrifugation at 1,000*g* to remove cell debris.

Apical cell surface proteins of $mCCD_{cl1}$ cells were gained by incubating cells grown on permeable supports for 30 min with 0.5 mg/ml EZ-linked sulfo-NHS-SS-Biotin in the apical compartment and with 100 mM glycine in the basolateral compartment. This was followed by a 30 min incubation with 100 mM glycine in both compartments to quench the remaining biotin. Cells were scraped, sonicated, and dissolved in isolation buffer (composition in millimolar: 250 sucrose, 10 Tris; pH 7.4) supplemented with protease inhibitors. Subsequently, membrane-enriched fractions were obtained by 15 min centrifugation at 4,000*g*, followed by an additional centrifugation step of the supernatant at 17,000*g* over 30 min. The pellet was dissolved in lysis buffer (composition in millimolar: 150 NaCl, 5 EDTA, 50 Tris; in %: 0.1 SDS, 0.5 sodium deoxycholate, 1 Nonidet P-40; pH 7.4) supplemented with protease inhibitors and mixed with Neutravidin beads (Pierce) to extract biotinylated proteins. After overnight incubation, beads were washed multiple times with the lysis buffer, high salt lysis buffer (supplemented with 500

mM NaCl), and salt-free lysis buffer (without NaCl and EDTA). Finally, the protein was separated from beads using reducing agent Rotiload (Carl Roth).

To prepare protein samples from mouse kidney tissue, the kidney cortex was dissected and homogenized in isolation buffer (composition in millimolar: 250 sucrose, 10 triethanolamin-HCl, 1.6 ethanolamine, 0.5 EDTA, pH 7.4; supplement with protease and phosphatase inhibitors) using a Dounce homogenizer. The total membrane fraction was obtained by sequential centrifugations of the homogenate at 1,000*g* and 20,000*g* for 15 and 30 min, respectively. The final pellet was resuspended in isolation buffer and passed through 23G and 27G needles.

To detect γ-ENaC cleavage fragments, 20 µg protein were deglycosylated with PNGase F according to the manufacturer's instructions (New England Biolabs). Subsequently, samples were boiled for 5 min at 95 °C and subjected to 10-12 % SDS-PAGE and western blot analysis. All western blot experiments were performed under reducing conditions. After separation, proteins were transferred to polyvinylidene difluoride membranes by semidry electroblotting and probed with mouse monoclonal antibodies against the serine protease domain of human TMPRSS2 (clone P5H9-A3; catalog no.: MABF2158; EMD Millipore Corp, validated against murine TMPRSS2, see Supplemental Figure 1) at a dilution of 1:2,500 or rabbit subunit-specific antibodies against murine α- (Pineda antibody service) or rat γ-ENaC (catalog no.: SPC-405; Stressmarq) at a dilution of 1:10,000 or 1:5,000, respectively. Horseradish peroxidase-labeled secondary goat anti-mouse (catalog no.: ab97023; Abcam) or goat-anti rabbit (catalog no.: G21234; Invitrogen) antibodies were used as secondary antibodies in a dilution of 1:50,000. To validate the separation of cell surface from intracellular proteins in biotinylation experiments, blots were stripped and reprobed using a polyclonal rabbit anti-β-actin antiserum (Sigma-Aldrich) at a dilution of 1:5,000. Densitometry was performed using ImageJ (National Institutes of Health). ATX Ponceau S (Fluka) membrane staining was used to control protein loading.

RNAscope stainings and immunohistochemistry

For RNAscope analysis, kidneys were perfusion-fixed in 10% Neutral Buffered Formalin, dehydrated in concentration ascending ethanol and isopropanol series and embedded in paraffin as described previously[49]. The hybridization protocol was performed on 5 µm tissue sections, pre-treated for 15 min with 1 × target retrieval solution and 15 min with protease plus reagent (contained in RNAscope kit). *Tmprss2* (mRNA) was detected using the RNAscope probe Mm-Tmprss2-C1 (1117611-C1). Multiplex RNAscope signals were detected using the AKOYA biosciences Opal™ fluorophore Opal570 (FP1488001KT) diluted in Tyramide Signal Amplification (TSA) buffer (1:750). Afterwards slices were washed with PBS, blocked for 60 min with 1% BSA in PBS and incubated with a polyclonal rabbit antibody directed against mouse β-ENaC[44] diluted in blocking solution overnight at 4 °C. Slices were incubated with fluorescent-labelled secondary antibodies (Anti-Rabbit-Alexa Fluor 488) and 4′,6-diamidino-2-phenylindole (DAPI) for 90 min at room temperature and mounted with ProLong™ Gold Antifade Mountant mounting medium (Thermo Fisher Scientific). Slides were stored at 4 °C until further use.

For analysis of tissue expression of γ-ENaC, kidneys were collected under control conditions or after 4 days on a low sodium diet. Paraffin-embedded formalin-fixed sections (2 µm) were deparaffinized with ethanol and rehydrated using standard protocols. Antigen retrieval was accomplished after heating for 5 min in antigen retrieval solution pH 6.1 (DAKO

Deutschland GmbH, Hamburg, Germany) using a pressure cooker (Rommelsbacher, Germany). Kidney sections were blocked with avidin and biotin for 15 min each, followed by blocking for another 30 minutes with normal goat serum diluted 1:5 in 50 mM tris(hydroxymethyl)-aminomethane (Tris), pH 7.4, supplemented with 5% (w/v) skim milk (Bio-Rad Laboratories, Munich, Germany). Sections were incubated overnight at 4 °C with an anti-γ-ENaC antibody (Stressmarq SPC-405, 1:200) and subsequent washing in Tris buffer (50 mM Tris, pH 7.4, supplemented with 0.05% (v/v) Tween 20 (Sigma-Aldrich, Munich, Germany; 3 x). The secondary antibody (a biotinylated goat anti-rabbit, Vector Laboratories, Burlingame, CA, USA; 1:500) was applied for 30 minutes at room temperature. Sections were further processed using the VectaStain ABC kit according to the manufacturer's instructions and DABImmpact (both Vector Laboratories) as substrate. Finally, the sections were counterstained in hemalaun, dehydrated, and mounted for observation using an Zeiss upright microscope.

Statistical methods

Statistical analysis was performed using GraphPad Prism, version 10.1.1 (GraphPad Software Inc.), and R environment for statistical computation (version 4.4.1, R Core Team; https://www.r-project.org/).

Supplemental Figures

Supplemental Figure 1

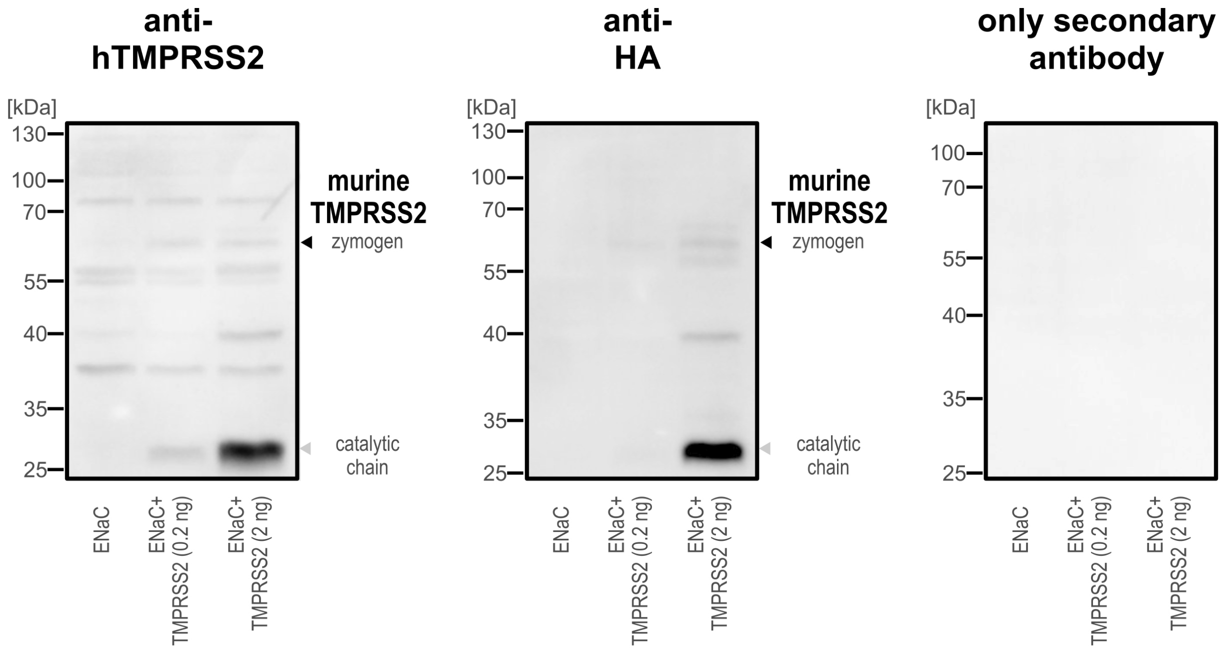

Supplemental Figure 1: Validation of anti-TMPRSS2 antibody in *X. laevis* oocytes.
Western blot analysis of TMPRSS2 expresssion in whole-cell lysates of *X. laevis* oocytes from one batch. Oocytes expressed ENaC alone (0.1 ng/subunit of injected cRNA) or co-expressed ENaC and 3'-HA tagged murine TMPRSS2 (0.2 ng or 2 ng of injected cRNA). Blots were stained either with a commercially available primary anti-human TMPRSS2 antibody (left panel), a primary anti-HA antibody (middle panel) or only a secondary goat anti-mouse antibody (right panel). The catalytic chain of TMPRSS2 and the zymogen form of TMPRSS2 are indicated by grey and black arrowheads, respectively. The sensitivity of the anti-hTMPRSS2 is probably higher than that of the anti-HA antibody. This probably explains why, with 0.2 ng of injected TMPRSS2 cRNA (second lane), a clear signal was detected with the anti-hTMPRSS2 but not with the anti-HA antibody.

Supplemental Figure 2

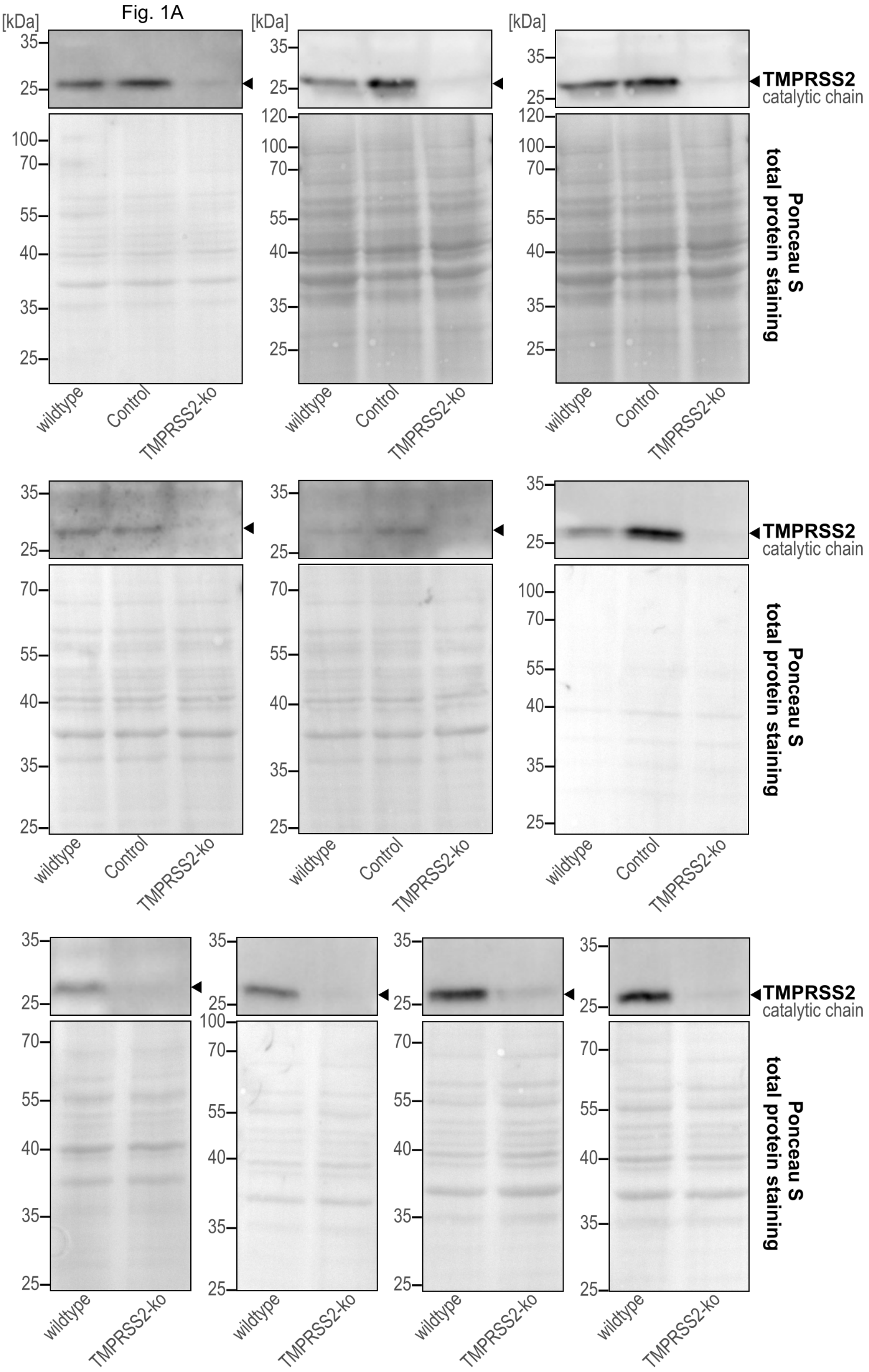

Supplemental Figure 2: Western blot detection of TMPRSS2 in $mCCD_{cl1}$ cells

Top panels: All western blots used to obtain summary data shown in Figure 1A (right panel) are depicted. The first blot is also shown in Figure 1A as representative. Expression of TMPRSS2 in whole cell lysates from $mCCD_{cl1}$ cells was analysed in wildtype, control and TMPRSS2-ko cells as indicated. Arrowheads indicate TMPRSS2 in its activated form (catalytic chain, ~26 kDa). *Bottom panels:* Ponceau S total protein staining for the same blot shown in the respective top panel confirmed similar protein loading in all lanes.

Supplemental Figure 3

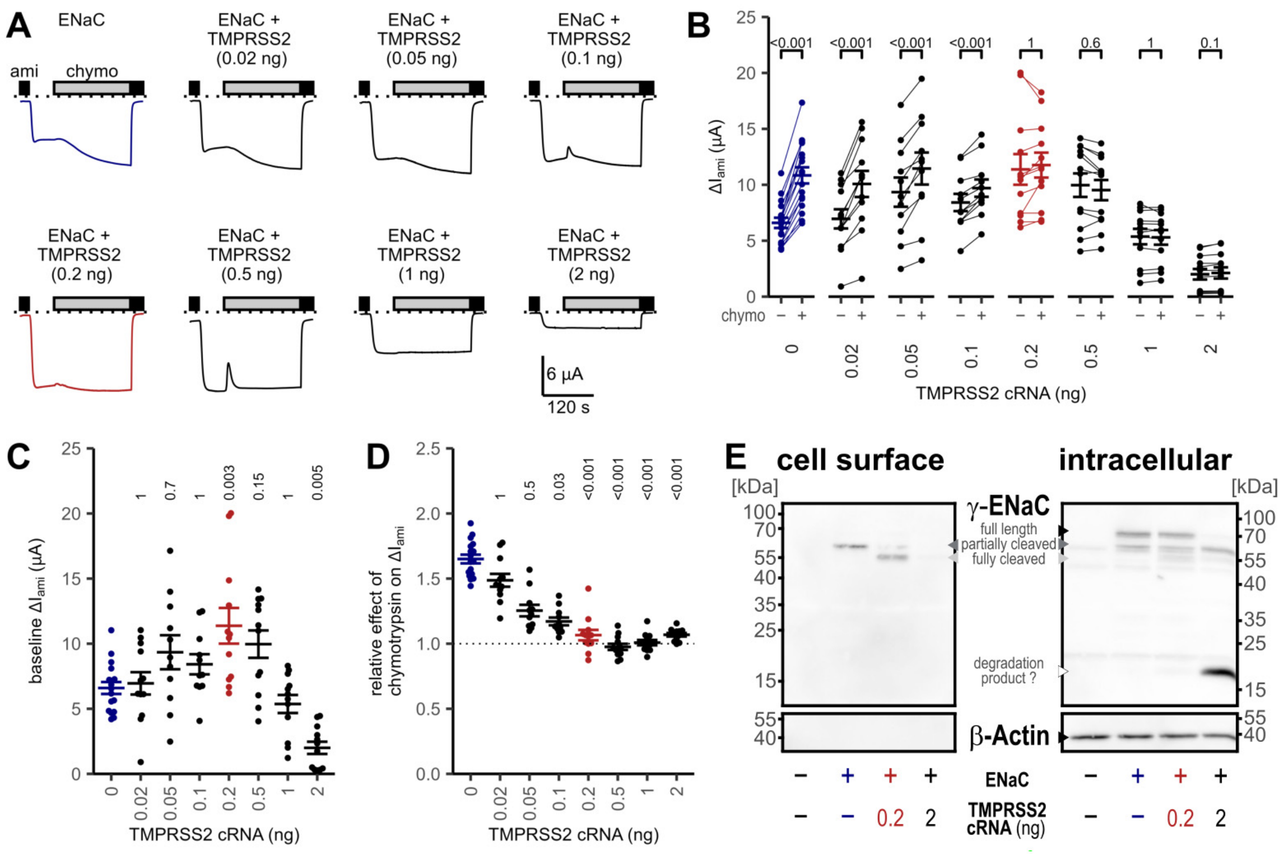

Supplemental Figure 3: Murine ENaC was proteolytically activated by coexpression of murine TMPRSS2 in *X. laevis* oocytes with a bell-shaped dependence of amiloride-sensitive ENaC-currents on the injected amount of TMPRSS2 cRNA

(A) Representative whole-cell current traces are shown from oocytes injected with cRNA for murine wildtype αβγENaC either alone or together with varying amounts of murine TMPRSS2 cRNA as indicated. Amiloride (ami, 2 µM) and chymotrypsin (chymo, 2 µg/ml) were present in the bath solution as indicated by black and grey bars, respectively. The dotted lines indicate zero current level. In oocytes expressing ENaC alone (recording marked in blue), washout of the ENaC-inhibitor amiloride revealed a Na^+ inward current component which could be further increased by ~1.7-fold by adding the prototypical serine protease chymotrypsin to the bath solution. In oocytes co-expressing ENaC and TMPRSS2 (0.2 ng cRNA; red trace), baseline amiloride sensitive currents were about twice as high as in control oocytes expressing ENaC alone, but were not further increased by chymotrypsin application. Interestingly, the stimulatory effect of TMPRSS2 on ENaC currents depended on the coinjected amount of TMPRSS2 cRNA in a bell-shaped manner. Thus, the stimulatory effect of TMPRSS2 co-expression increased with increasing amounts of injected TMPRSS2 cRNA until a maximum was reached with 0.2 ng/oocyte, the amount we routinely used in our experiments. A further increase in cRNA resulted in a substantial decline of ENaC currents even below the level observed without TMPRSS2 coexpression. This decline of ENaC currents with high amounts of TMPRSS2 cRNA was probably caused by unspecific effects like proteolytic degradation of ENaC due to overexpression of TMPRSS2. This most likely explains previously reported findings of ENaC inhibition by TMPRSS2 co-expression [Donaldson SH, Hirsh A, Li DC, et al. Regulation of the epithelial sodium channel by serine proteases in human airways. *J Biol Chem*. 2002; 277(10):8338-8345. doi:10.1074/jbc.M105044200] which have led to the erroneous conclusion that TMPRSS2 does not proteolytically activate ENaC. In contrast, our previously published data convincingly demonstrated a clear stimulatory effect of TMPRSS2 coexpression on ENaC due to proteolytic channel activation by γ-ENaC cleavage[28]. As demonstrated here, this is not limited to human but also applies to murine TMPRSS2 and ENaC orthologues.

(B) ENaC-mediated amiloride-sensitive whole-cell currents (ΔI_{ami}) were determined from similar experiments as shown in (*A*) in the absence (−) or presence (+) of chymotrypsin. Lines connect data points from an individual oocyte. Mean ± SEM and individual datapoints are shown; Two-sided paired Student's

t-test with Bonferroni correction for multiple testing (n=11-17, N=2-3; N indicates the number of different oocyte batches, and n indicates the number of individual experiments per experimental group).

(C) Summary data of baseline ENaC-mediated currents in the absence of chymotrypsin (ΔI_{ami}) obtained in the same experiments as shown in (*B*). ANOVA (p-value <0.001) with Bonferroni post-hoc test was used to calculate p-values for comparisons with the baseline current values obtained in oocytes expressing only ENaC (0 ng TMPRSS2 cRNA).

(D) Relative stimulatory effect of chymotrypsin on ΔI_{ami} summarised from data shown in (*B*). The dotted line indicates a relative effect of one (no effect). Mean ± SEM and data points for individual oocytes are shown; Kruskal-Wallis (p-value <0.001) with Dunn's Test for Multiple Comparisons of log-transformed values was used to calculate p-values for comparisons with the values obtained in oocytes expressing only ENaC (0 ng TMPRSS2 cRNA).

(E) Western blots showing cell surface (*left upper panel*) or intracellular (*right upper panel*) expression of γ-ENaC in oocytes from one batch injected with cRNA for αβγENaC without (−) or with 0.2 ng or 2 ng cRNA encoding for murine TMPRSS2. Non-injected oocytes served as a control (first lane). Full length (~70 kDa), partially cleaved (~60 kDa), and fully cleaved (~55 kDa) γ-ENaC are indicated by black, dark grey, or light grey arrowheads, respectively. As previously reported, the stimulatory effect of chymotrypsin is due to full proteolytic cleavage of partially cleaved γ-ENaC present at the oocyte cell surface[19,21,28]. Accordingly, TMPRSS2 co-expression converted partially cleaved γ-ENaC (~60 kDa) at the cell surface into its fully cleaved form (~55 kDa). An additional band at ~17 kDa appeared with the high amount of TMPRSS2 cRNA and may represent a degradation product of γ-ENaC. Blots were stripped and reprobed using an antibody against β-actin (*lower panels*) to validate the separation of cell surface proteins from intracellular proteins.

Supplemental Figure 4

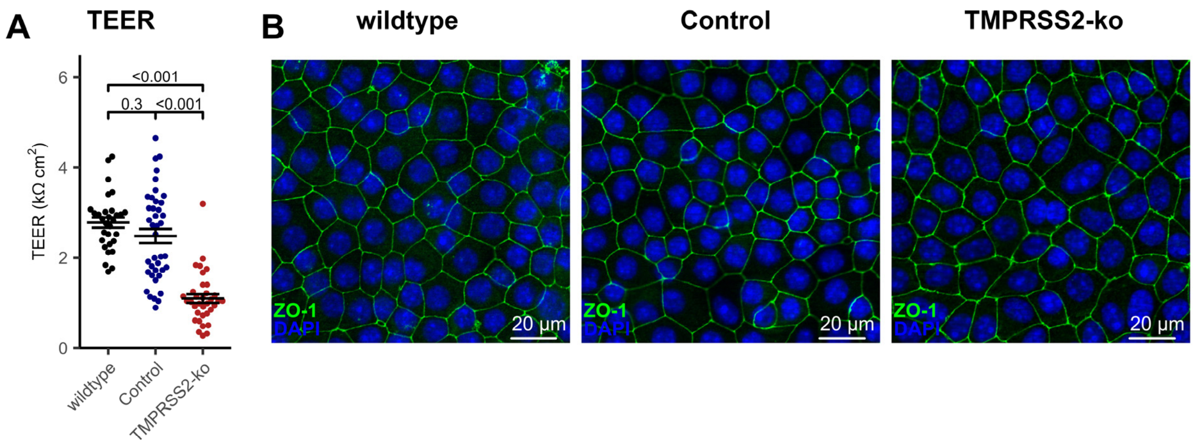

Supplemental Figure 4: TMPRSS2 knockout in mCCD$_{cl1}$ cells did not affect normal epithelial monolayer formation in culture.

(A) Transepithelial electrical resistance (TEER) values recorded at the beginning of Ussing chamber recordings shown in Figure 1, 2, and Supplemental Figure 6. Mean ± SEM and individual data points are shown; One-way ANOVA (p-value <0.001) with Bonfrerroni post hoc test was used to calculate p-values (wildtype: n=30, non-targeting control: n=40, TMPRSS2-ko: n=35).

(B) Representative immunfluorescence staining for the tight junction Zonula occludens-1 protein (ZO-1, in green) in wildtype, non-targeting control and TMPRSS2-knockout mCCD$_{cl1}$ cells. Nuclei are stained with DAPI. Monolayers of mCCD$_{cl1}$ cells were fixed on permeable supports with 4 % paraformaldehyde in PBS, permeabilized with 0.1 % Triton X-100, and blocked with Roti Immunoblock (Carl Roth). Cell preparations were stained by overnight incubation with mouse monoclonal anti-ZO-1 (zonula occludens protein 1) antibody (clone ZO1-1A12; Invitrogen), at a dilution of 1:250 dissolved in PBS, supplemented with 0.5% bovine serum albumin and 0.04% sodium azide. After subsequent incubation for 1 h with a goat anti-mouse DyLight488 antibody (Thermo Fisher Scientific) at a dilution of 1:400, cells were mounted on slides using DAPI mounting medium (Fluoroshield with DAPI, Sigma-Aldrich), and images were acquired using a ZEISS microscope (Axiovert 200M) and apotome technology.

Supplemental Figure 5

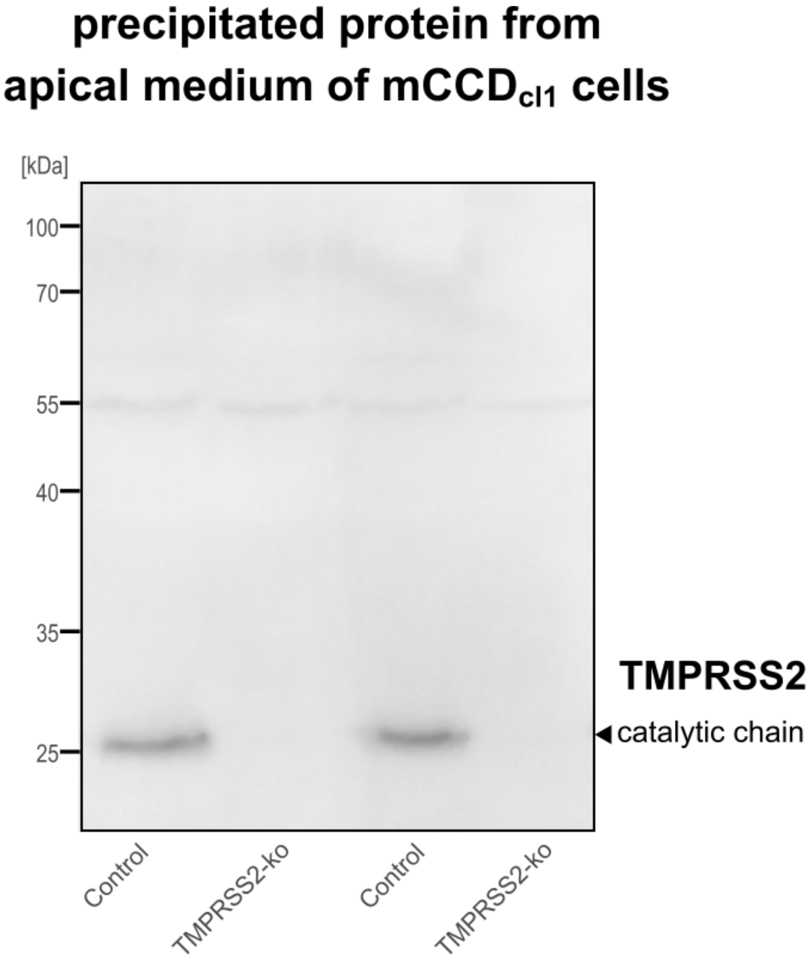

Supplemental Figure 5: TMPRSS2 was present in the apical medium of $mCCD_{cl1}$ cells.
TMPRSS2 was detected by western blot analysis in cell culture medium taken from the apical compartment of non-targeting control (Control), or TMPRSS2-knockout (TMPRSS2-ko) $mCCD_{cl1}$ cells. A black arrowhead indicates TMPRSS2 in its activated (catalytic chain, ~26 kDa) form.

Supplemental Figure 6

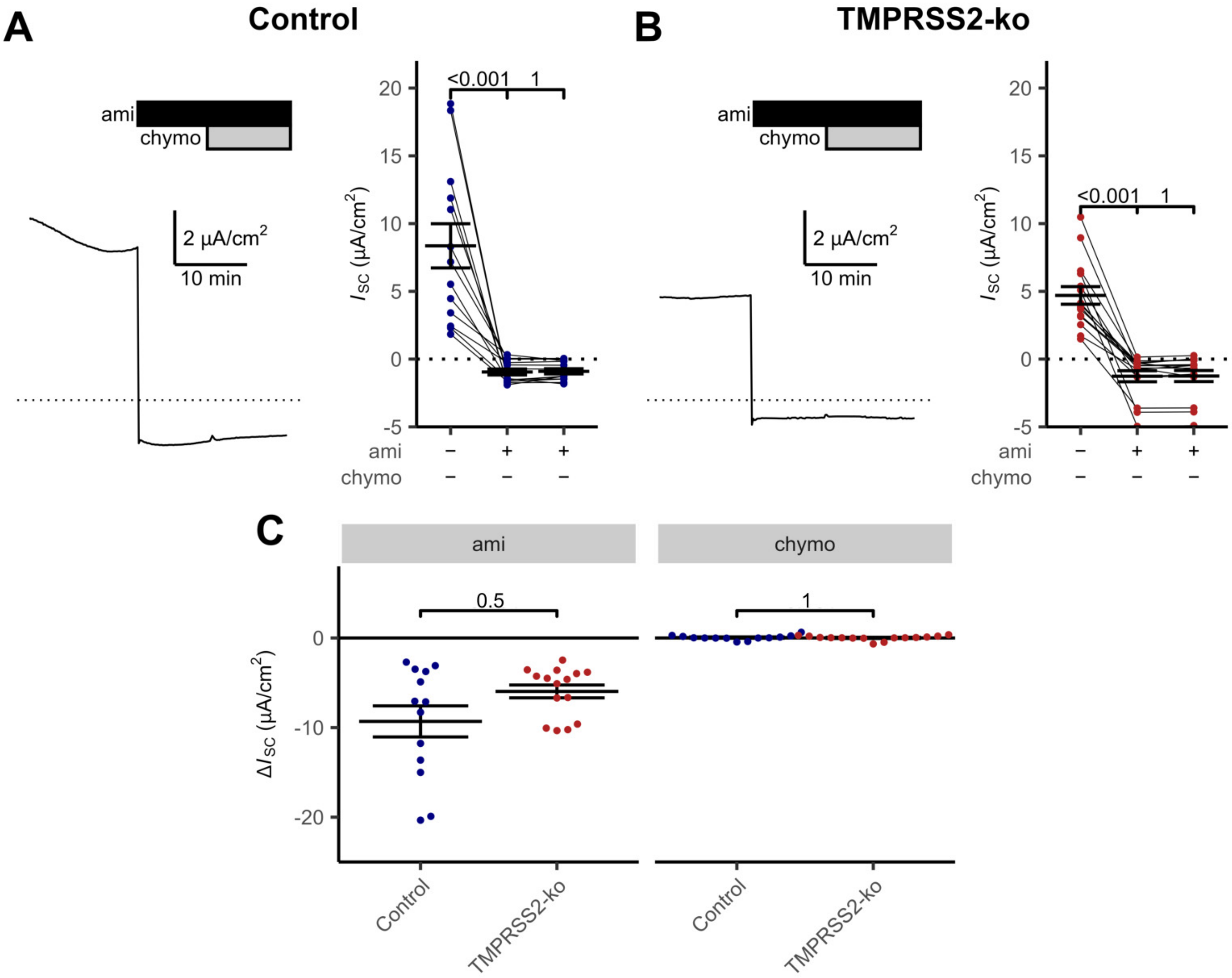

Supplemental Figure 6: Chymotrypsin failed to stimulate ENaC in the presence of amiloride in TMPRSS2-knockout and control mCCD$_{cl1}$ cells

(A,B) *Left panels:* Representative equivalent short circuit current (I_{SC}) traces recorded from non-targeting control (*A*), or TMPRSS2-knockout mCCD$_{cl1}$ cells (*B*) are shown. Amiloride (ami, 10 µM) and chymotrypsin (chymo, 20 µg/ml) were present in the bath solution as indicated by black and grey bars, respectively. The dotted lines indicate zero current levels. *Right panels:* Plots summarize data from similar experiments as shown in the corresponding left panels. Values were calculated as described in Figure 1C. Values obtained in the same measurement are connected with a line. Mean ± SEM and data points for individual permeable supports are shown. A: n=13, B: n=15. Kruskal-Wallis (*p*-value <0.001 (Control), <0.001 (TMPRSS2-ko)) with Dunn's Test for multiple comparisons.

(C) Summary data from the same experiments as shown in (*A, B*). ΔI_{SC} values were calculated essentially as described in Figure 1E. Mean ± SEM and data points for individual measurements are shown for the effect of amiloride (ami) and for the effect of chymotrypsin applied in the presence of amiloride (chymo). Two-sided Wilcoxon Signed Rank test with Bonferroni correction for multiple testing.

Supplemental Figure 7

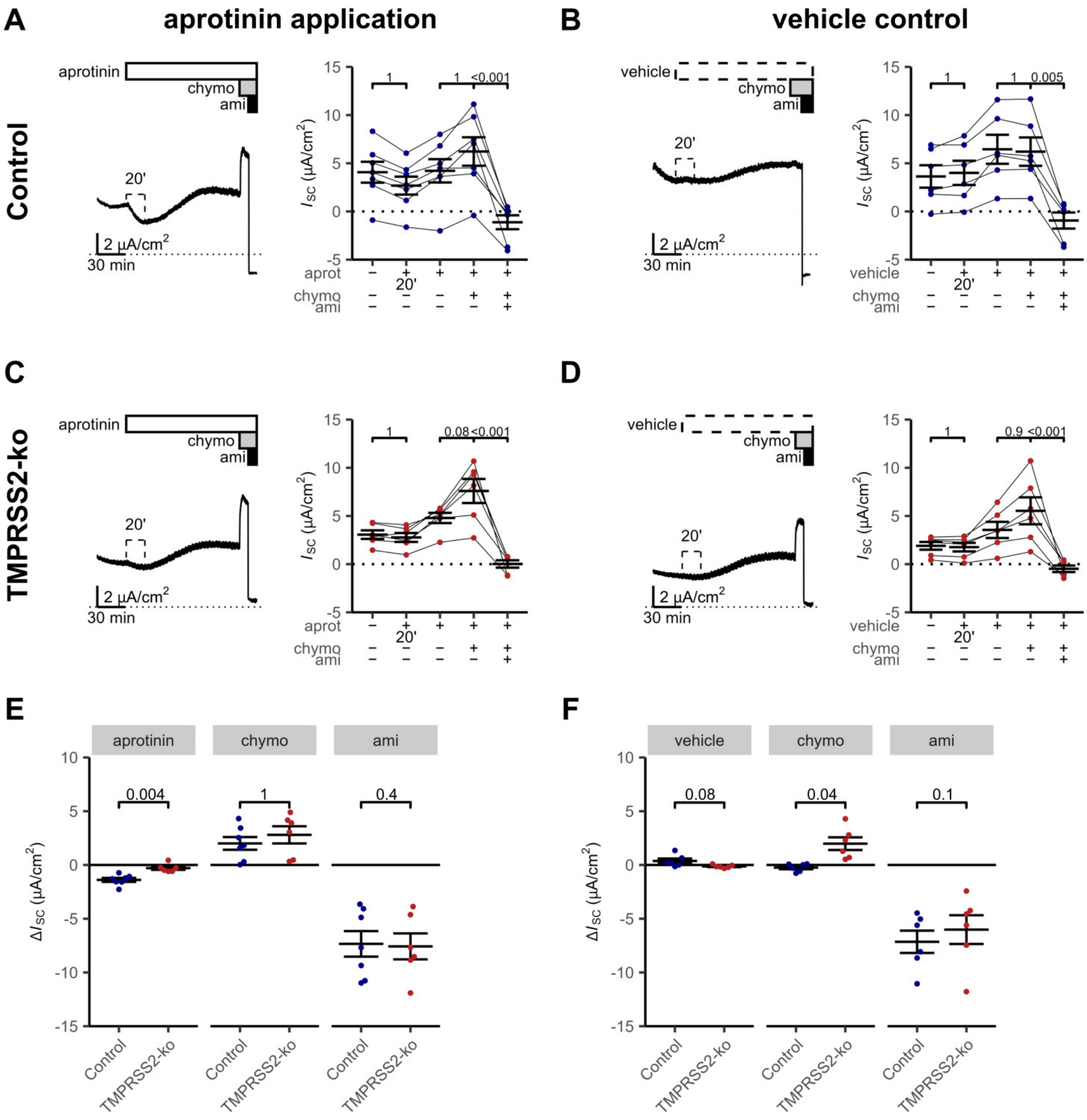

Supplemental Figure 7: Apically applied aprotinin reduced I_{SC} in control but not in TMPRSS2-ko mCCD$_{cl1}$ cells.

(A-D) *Left panels:* Representative equivalent short circuit current (I_{SC}) traces recorded from non-targeting control (*A, B*), or TMPRSS2-knockout mCCD$_{cl1}$ cells (*C, D*) are shown. Aprotinin (30 µg/ml), amiloride (ami, 10 µM) and chymotrypsin (chymo, 20 µg/ml) were present in the bath solution as indicated by bars in white, black and grey, respectively. A white bar with a dashed line indicates vehicle application (0.9% NaCl) in *B* and *D*. The dotted lines indicate zero current levels. A dashed line indicates a time interval of 20 minutes after application of aprotinin or vehicle. *Right panels:* Plots summarize data from similar experiments as shown in left panels. Values were measured immediately before and 20 minutes after application of aprotinin or vehicle, immediately before application of chymotrypsin, immediately before application of amiloride, and at the end of the experiment. Values obtained in the same recording are connected with a line. Mean ± SEM and data points for individual permeable supports are shown. n=6-7. ANOVA (*p*-values 0.001 (*A*), 0.002 (*B*), <0.001 (*C*), <0.001 (*D*)) with Bonferroni post-hoc test.

(E, F) Summary data from the same experiments as shown in (*A-D*). ΔI_{SC} values for aprotinin (*E*) or vehicle (*F*) application were calculated by subtracting I_{SC} values obtained immediately before application of aprotinin or vehicle from values reached 20 minutes after application of these compounds (timepoint marked by dashed lines *A-D*). ΔI_{SC} values for chymotrypsin and amiloride application were calculated essentially as described in Figure 1E. Mean ± SEM and data points for individual measurements are shown. Two-sided unpaired Student's *t*-test with Bonferroni correction for multiple testing.

Supplemental Figure 8

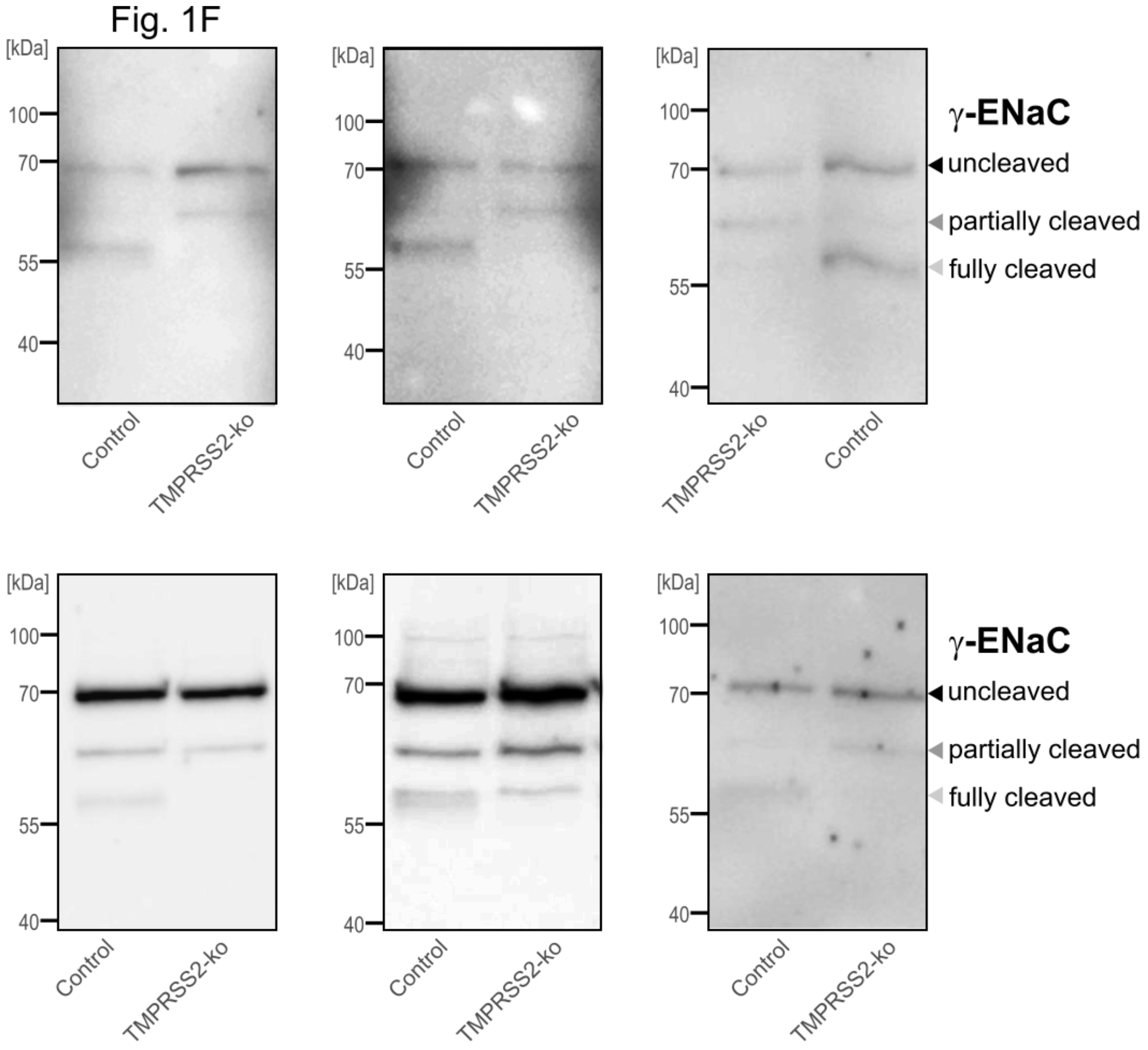

Supplemental Figure 8: Western blot detection of γ-ENaC cleavage fragments in mCCD$_{cl1}$ cells
All western blots used to obtain summary data shown in Figure 1F (right panel) are depicted. The first blot is also shown in Figure 1F as representative. Expression of γ-ENaC in cell surface fractions from mCCD$_{cl1}$ cells was analysed in TMPRSS2-ko and control cells as indicated. Uncleaved (~70 kDa), partially cleaved (~60 kDa), and fully cleaved (~55 kDa) γ-ENaC are indicated by black, dark grey, and light grey arrowheads, respectively.

Supplemental Figure 9

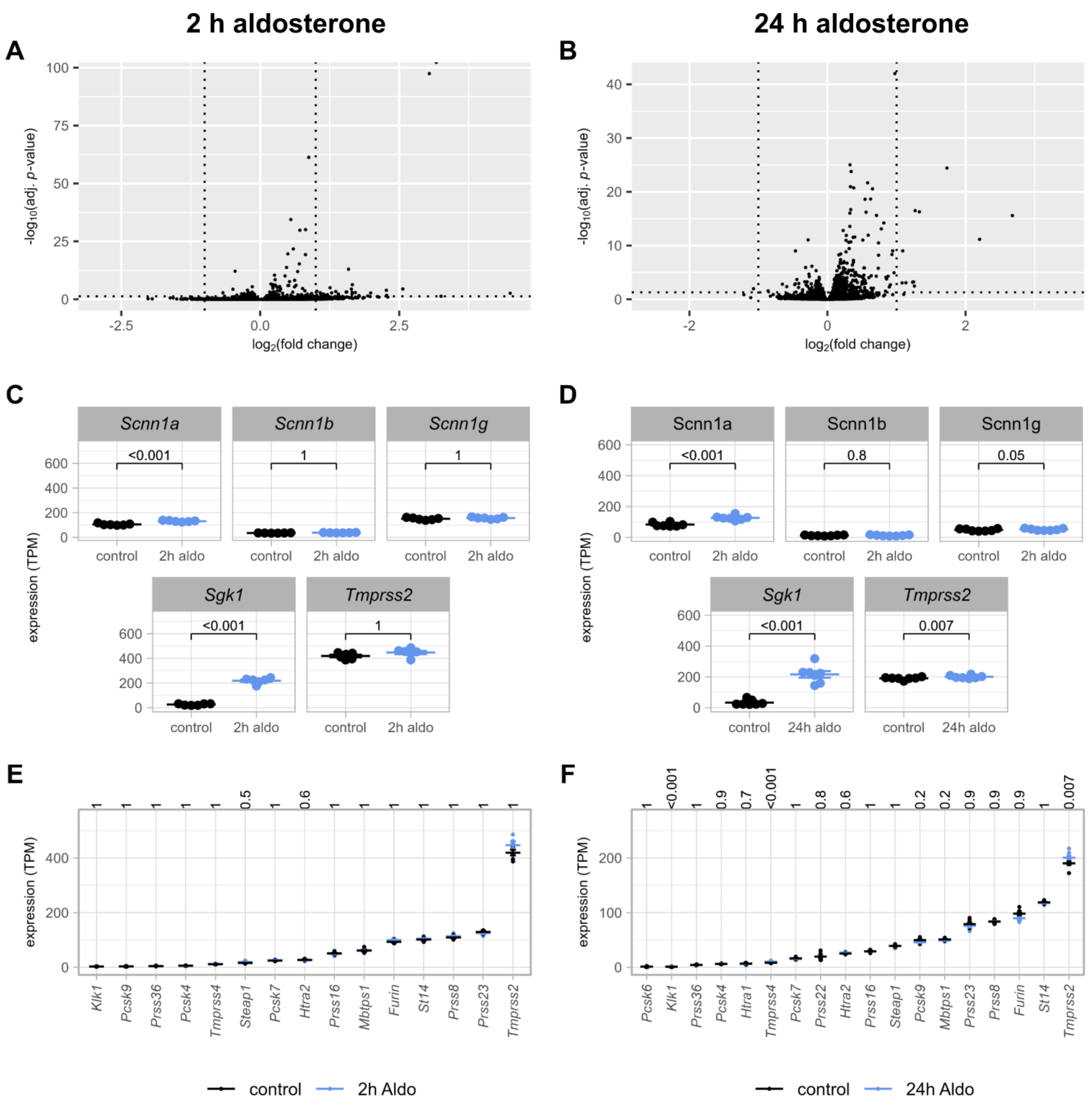

Supplemental Figure 9: RNA-sequencing analysis of mCCD$_{cl1}$ cells treated over 2 h or 24 h with aldosterone

(A, B) Vulcano plot of differentially expressed genes in mCCD$_{cl1}$ cells treated for 2 h (*A*) or 24 h (*B*) with 3 nM aldosterone. Aldosterone treated cells were compared with vehicle (0.9% NaCl) treated control cells. Negative $\log_{10}$ of the adjusted *p*-values over the $\log_2$ of the fold change in expression (aldosterone-treated / control) are plotted. Protein coding gene transcripts with at least 2-fold change in expression (vertical dotted lines) and an adjusted *p*-value <0.05 (horizontal dotted lines) are listed in Supplemental Tables S1 and S2.

(C-F) Expression level in transcripts per million (TPM) for selected genes as indicated (*C, D*) or genes encoding serine proteases with an expression of at least 1 TPM (*E, F*) in control (black) *vs.* aldosterone-treated cells (light blue, 2 h in *C, E* or 24 h in *D, F*) are shown. Genes encoding serine proteases, including type II transmembrane serine proteases, kallikreins and proprotein convertases of subtilisin/kexin gene family, were selected according to the classification provided by the HUGO Gene Nomenclature Committee (HGNC). Mitochondrial proteases were excluded from the analysis. Transcriptomic upregulation of *Scnn1a* and *Sgk1* by aldosterone was in line with previous reports[51,54,55]. Mean ± SEM and individual datapoints from individual permeable supports are shown together with the corresponding adjusted *p*-values.

Supplemental Figure 10

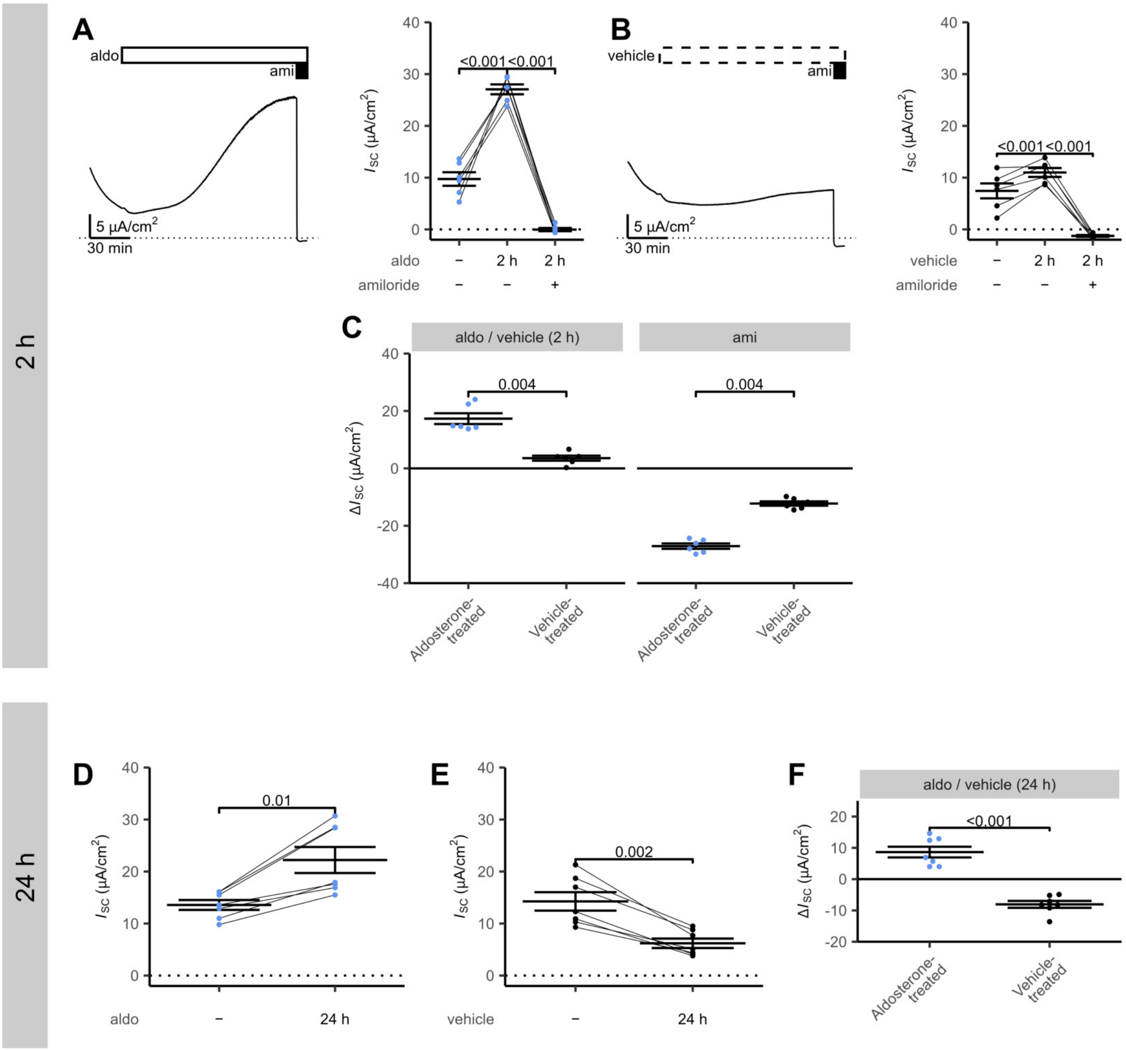

Supplemental Figure 10: Control I_{SC} recordings in mCCD$_{cl1}$ cells used for RNA-sequencing analysis.

(A, B) *Left panels:* Representative equivalent short circuit current (I_{SC}) recordings from wildtype mCCD$_{cl1}$ cells are shown. In (*A*) aldosterone (3 nM) was present in the apical and basolateral bath solution during the time period indicated by a white bar with a continuous outline. In (*B*) a corresponding vehicle (0.9% NaCl) was present as indicated by a white bar with a dashed outline. Amiloride (ami, 10 µM) was present in the apical bath solution as indicated by black bars. The dotted lines indicate zero current levels. *Right panels:* Plots summarize data from similar experiments as shown in left panels. Values were measured immediately before application of aldosterone or vehicle, immediately before application of amiloride, and at the end of the experiment. Values obtained in the same measurement are connected with a line. Mean ± SEM and data points for individual permeable supports are shown. n=6. ANOVA (*p*-values <0.001 (*A*), <0.001 (*B*)) with Bonferroni post-hoc test.

(C) Summary data from the same experiments as shown in (*A, B*). ΔI_{SC} values were calculated essentially as described in Figure 1E. Mean ± SEM and data points for individual measurements are shown. Two-sided Wilcoxon Signed Rank test with Bonferroni correction for multiple testing.

(D, E) I_{SC} was assessed by spot measurements of transepithelial resistance and transepithelial potential difference before (−) and after 24 h application of aldosterone (*D*) or vehicle (*E*). Values obtained in the same measurement are connected with a line. Mean ± SEM and data points for individual permeable supports are shown (n=6). Two-sided Wilcoxon Signed Rank test with Bonferroni correction for multiple testing.

(F) Summary data from the same experiments as shown in (*D*, *E*). ΔI_{SC} values were calculated by subtracting values reached after 24 h application of aldosterone or vehicle from baseline values before application. Mean ± SEM and data points for individual measurements are shown. Two-sided Wilcoxon Signed Rank Test.

Supplemental Figure 11

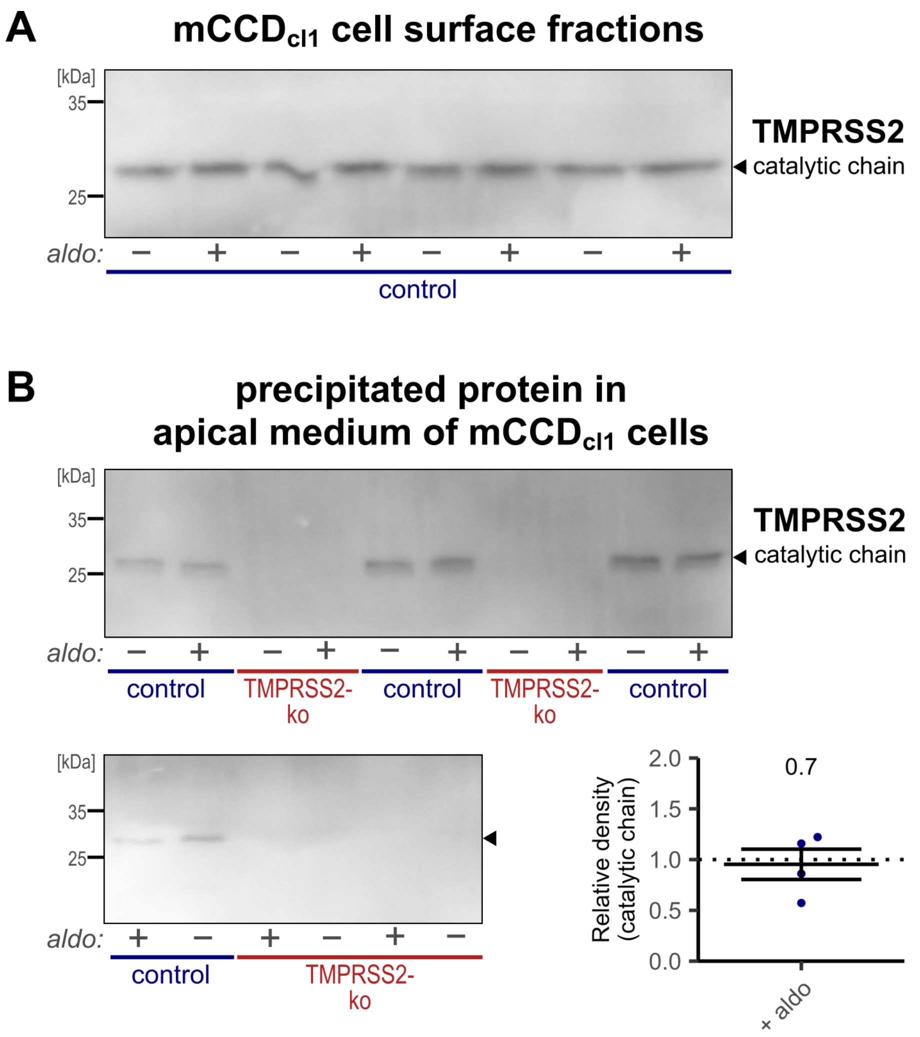

Supplemental Figure 11: Aldosterone did not increase TMPRSS2 abundance at the cell surface or in apical medium of control mCCD$_{cl1}$ cells.

(A) Western blot analysis of the apical cell-surface fraction of non-targeting control mCCD$_{cl1}$ cells to assess the effect of aldosterone on TMPRSS2 expression. Prior to harvesting, cells were maintained for 3 hours in the presence (+) or absence (−) of 3 nM aldosterone (aldo) as indicated. A black arrowhead indicates TMPRSS2 in its activated (catalytic chain, ~26 kDa) form. Densitometric evaluation of this blot was included in the summary data shown in Figure 2D (right panel).

(B) TMPRSS2 was detected by western blot analysis in cell culture medium taken from the apical compartment of non-targeting control (control, blue), or TMPRSS2-knockout (TMPRSS2-ko, red) mCCD$_{cl1}$ cells. Prior to harvesting, medium was exchanged and cells were maintained for 3 hours in the presence (+) or absence (−) of 3 nM aldosterone (aldo) as indicated. A black arrowhead indicates TMPRSS2 in its activated (catalytic chain, ~26 kDa) form. *Inset:* Densitometric evaluation of western blots from control cells was performed as described in Figure 2D (right panel).

Supplemental Figure 12

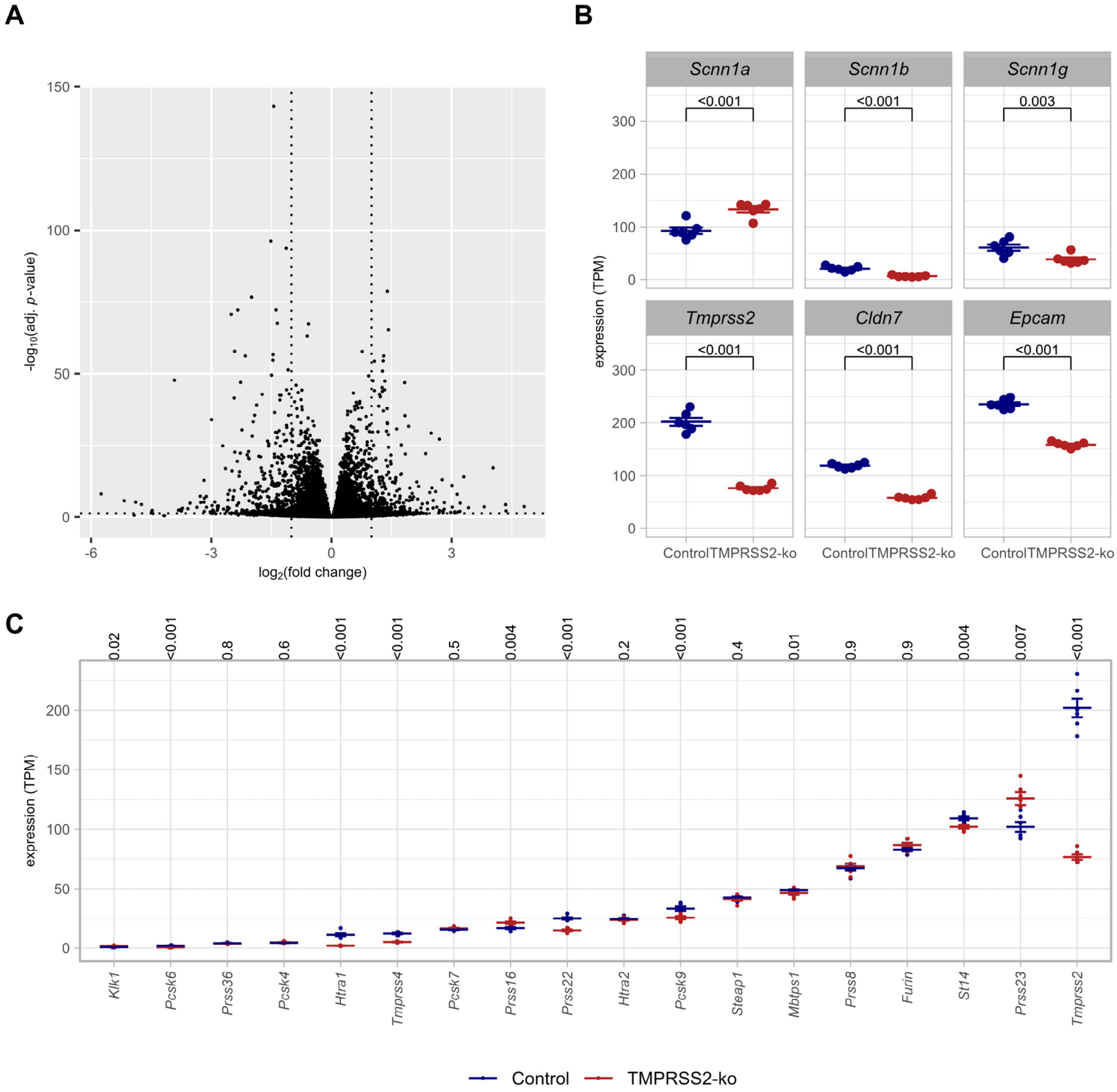

Supplemental Figure 12: RNA sequencing analysis of Control and TMPRSS2-ko mCCD$_{cl1}$ cells.

(A) Vulcano plot of differentially expressed genes in TMPRSS2-ko compared with non-targeting control cells plotting negative $\log_{10}$ of the adjusted *p*-values over the $\log_2$ of the fold changes in expression (TMPRSS2-ko / Control). Dotted lines mark borders for transcripts with at least 2-fold change in expression and an adjusted *p*-value <0.05. The 20 transcripts with the highest up- and downregulation are listed in Supplemental Table 3.

(B, C) Expression level in transcripts per million (TPM) for selected genes as indicated (*B*) or genes encoding serine proteases detected with an expression of at least 1 TPM (*C*) in control (dark blue) *vs.* TMPRSS2-ko cells (red), respectively. Genes encoding serine proteases, including type II transmembrane serine proteases, kallikreins and proprotein convertases of subtilisin/kexin gene family, were selected according to the classification provided by the HUGO Gene Nomenclature Committee (HGNC). Mitochondrial proteases were excluded from the analysis. Mean ± SEM and individual datapoints from individual permeable supports are shown. Downregulation of *Cldn7* and *Epcam* in TMPRSS2-ko cells is consistent with previous observations[34] and the reduced transepithelial resistances in these cells as shown in Supplemental Figure 4A. Like Kim *et al.*, who did not observe a compensatory transcriptional upregulation of proteases in *Tmprss2*$^{-/-}$ mice[46], we did not find any relevant transcriptional upregulation of other highly-expressed proteases in TMPRSS2-deficient mCCD$_{cl1}$ cells except for a slightly increased expression of *Prss23*.

Supplemental Figure 13

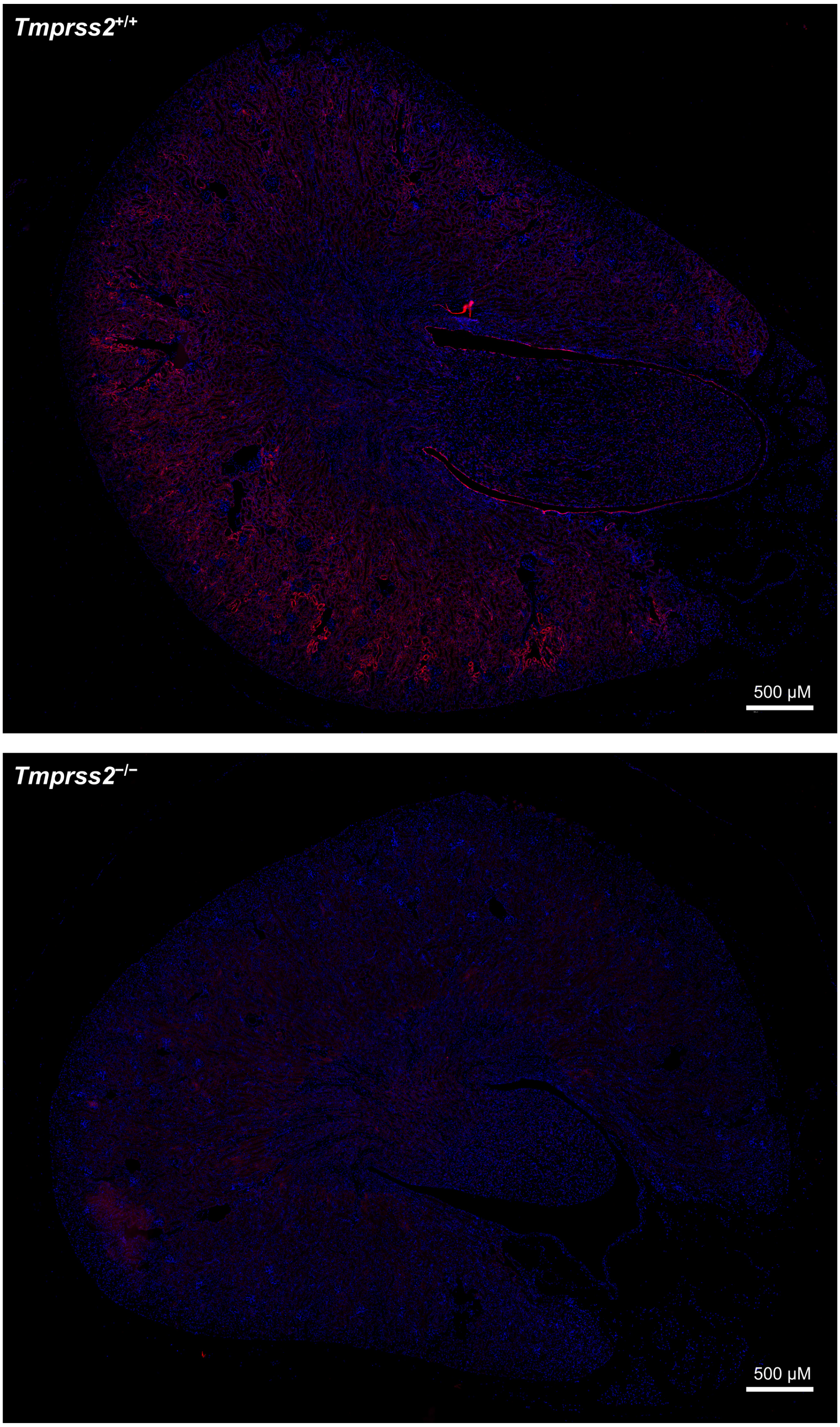

Supplemental Figure 13: Confirmation of TMPRSS2 knockout in *Tmprss2*$^{-/-}$ kidneys.
Representative microscopic images of mouse kidney slices of *Tmprss2*$^{+/+}$ *(top panel)* and *Tmprss2*$^{-/-}$ *(bottom panel)* mice are shown. *Tmprss2* mRNA signal (red) was obtained using RNAscope technique and merged with nuclear DAPI-staining (blue).

Supplemental Figure 14

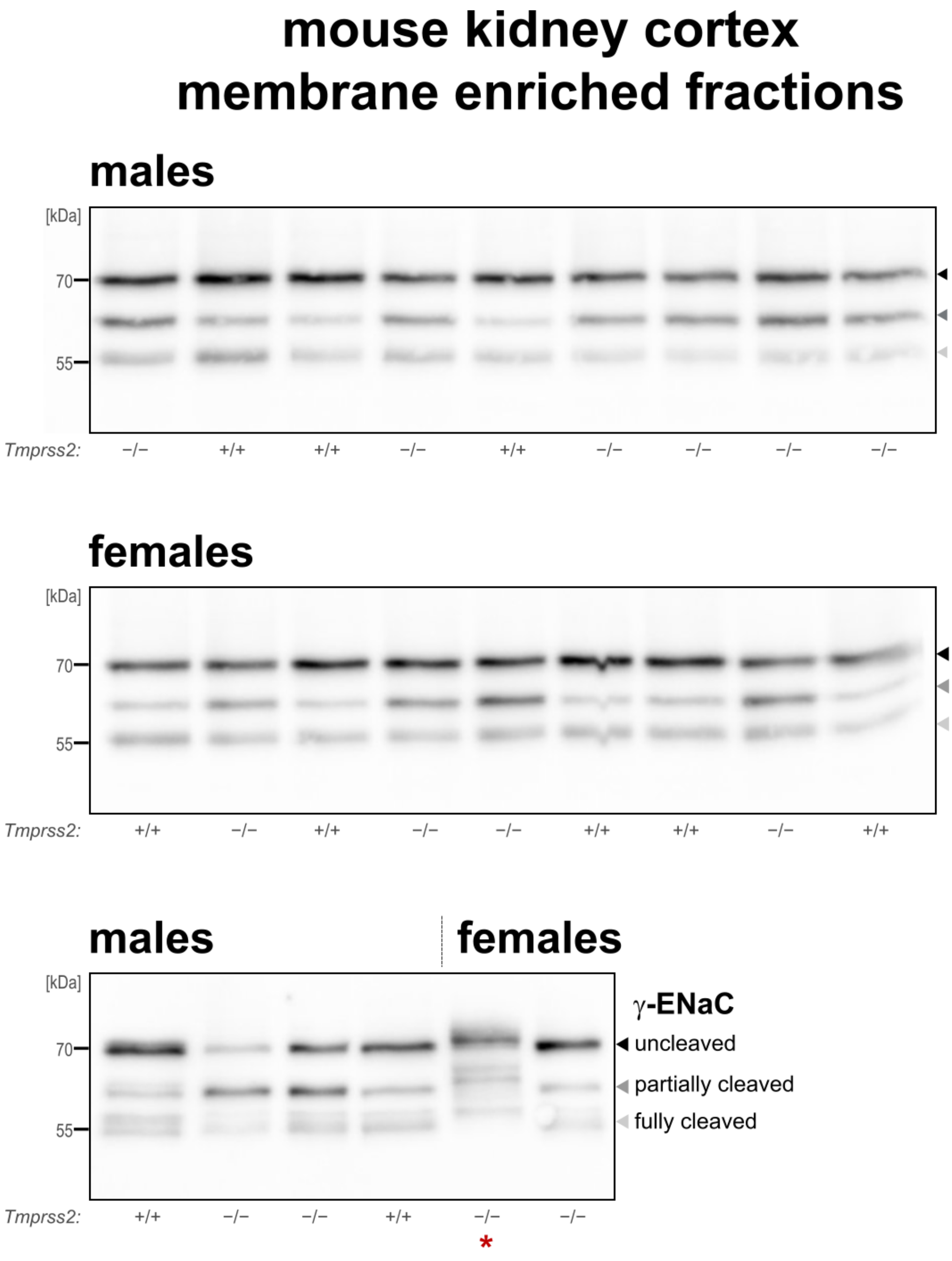

Supplemental Figure 14: Original western blots of γ-ENaC in mouse kidney cortex. Original images of the same western blots as used for Figure 4 are shown with original lane order. Expression of γ-ENaC in membrane enriched fractions from mouse kidney cortex was analysed in *Tmprss2*$^{+/+}$ (+/+) and *Tmprss2*$^{-/-}$ mice (−/−) as indicated. Uncleaved (~70 kDa), partially cleaved (~60 kDa), and fully cleaved (~55 kDa) γ-ENaC are indicated by black, dark grey, and light grey arrowheads, respectively. A red asterisk marks a lane with shifted signals (technical artifact) that was not included in data analysis shown in Figure 4.

Supplemental Figure 15

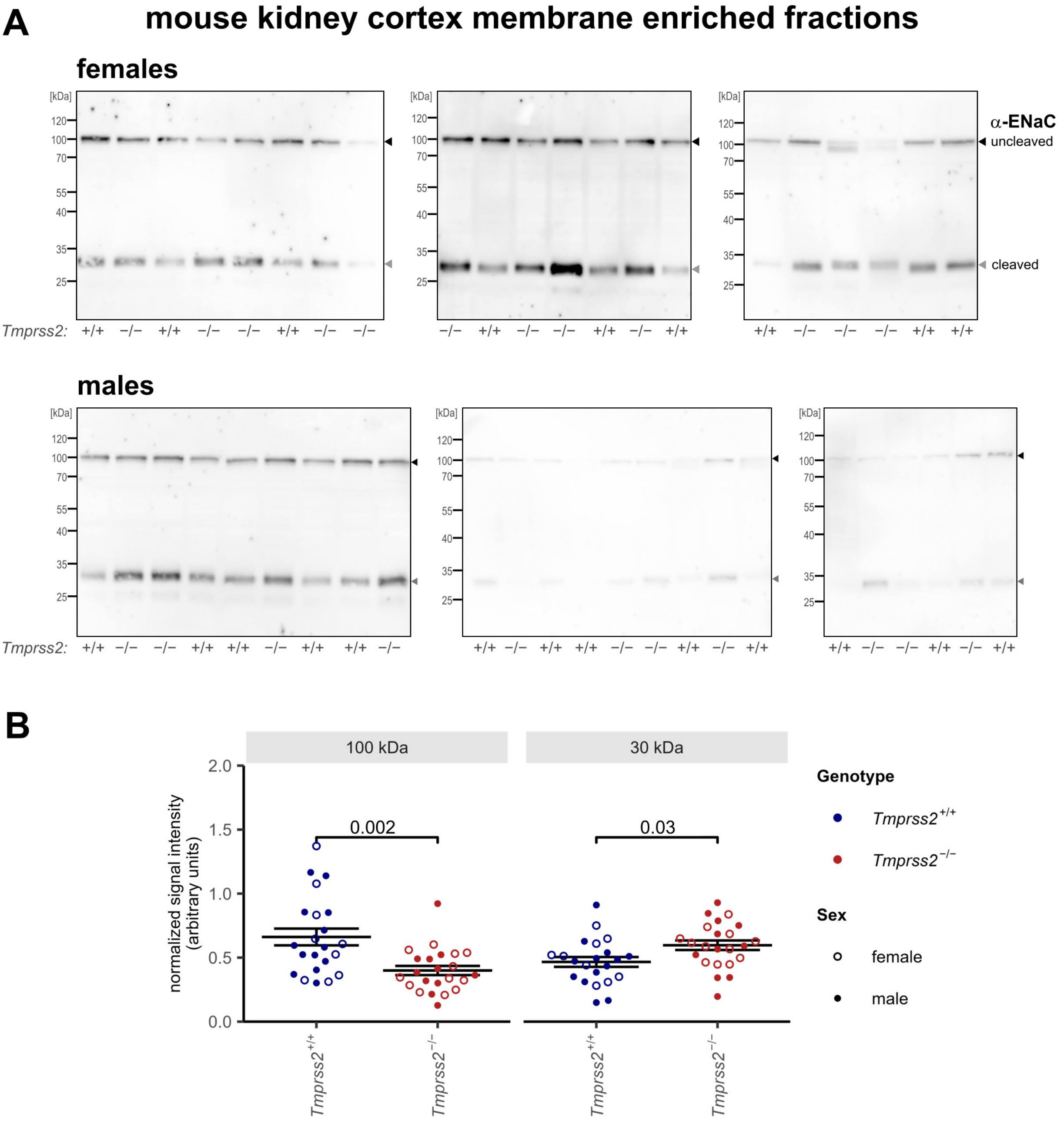

Supplemental Figure 15: In *Tmprss2*$^{-/-}$ mice, proteolytic processing of renal α-ENaC was not reduced but rather increased in comparison with *Tmprss2*$^{+/+}$ mice

- **(A)** Western blots showing expression of endogenous α-ENaC in membrane enriched fractions from mouse kidney cortex. Uncleaved (~100 kDa) α-ENaC is indicated with a black arrowhead, signals corresponding to cleaved (~30 kDa) α-ENaC fragments are indicated with grey arrowheads.
- **(B)** Densitometric evaluation of western blots shown in (*A*). The densitometric α-ENaC signal in each lane was normalized to the Ponceau S total protein staining from the same lane. Mean ± SEM and data points for individual western blots are shown. Data points from female and male mice are represented with open and closed symbols, respectively. Wilcoxon Signed Rank Test with Bonferroni correction for multiple testing.

Supplemental Figure 16

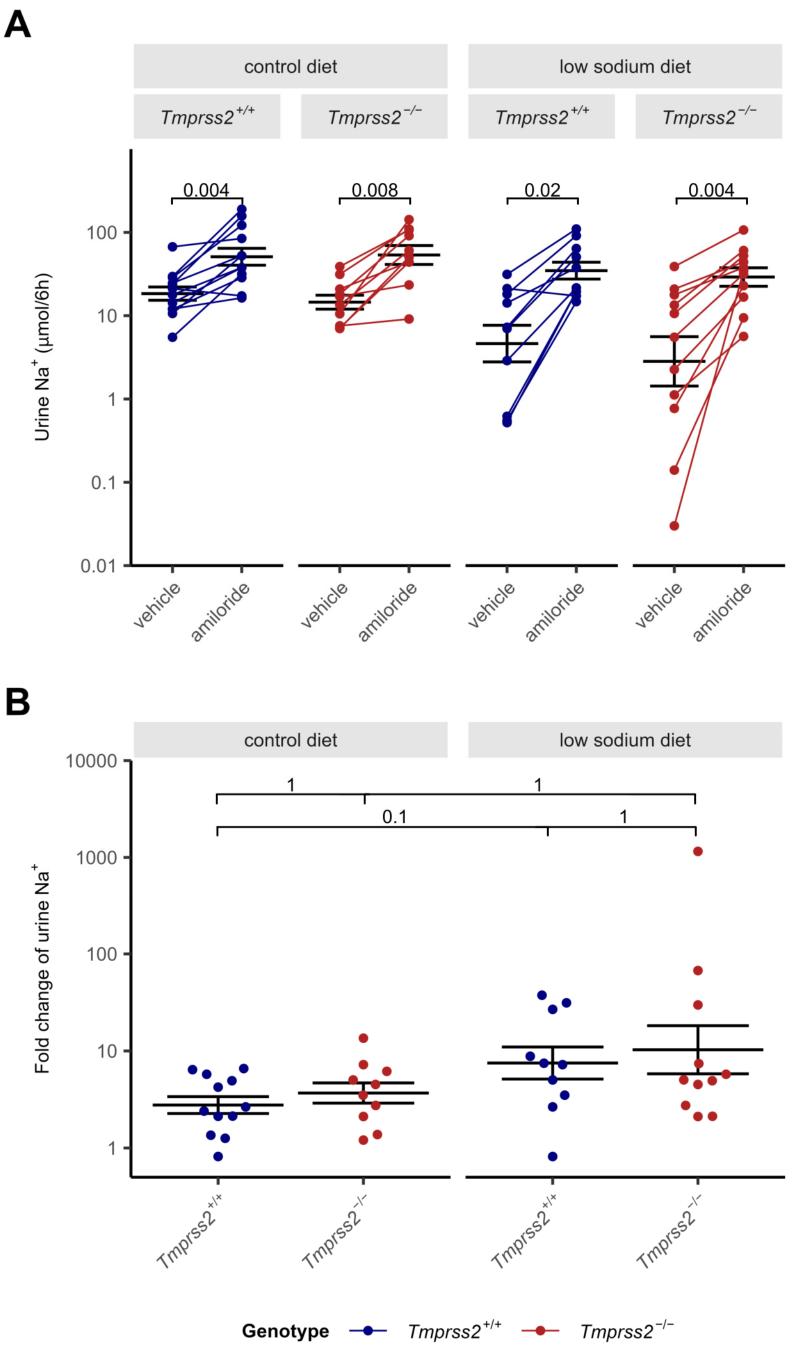

Supplemental Figure 16: Natriuretic response to amiloride is preserved in *Tmprss2*$^{-/-}$ mice

(A) Natriuretic response expressed as urinary sodium excretion over 6 hours after acute administration of vehicle (injectable water, 5 µl/gBW) or the ENaC inhibitor amiloride (10 µg/gBW) under a control or low sodium diet. Mean ± SEM and individual datapoints (n=10-12) are shown. Two-sided paired Wilcoxon Signed Rank Test with Bonferroni correction for multiple testing.

(B) Fold change of the natriuretic response, reflecting the slope from (*A*). Kruskal Wallis with Dunn's Multiple Comparisons Test of log-transformed values.

Supplemental Figure 17

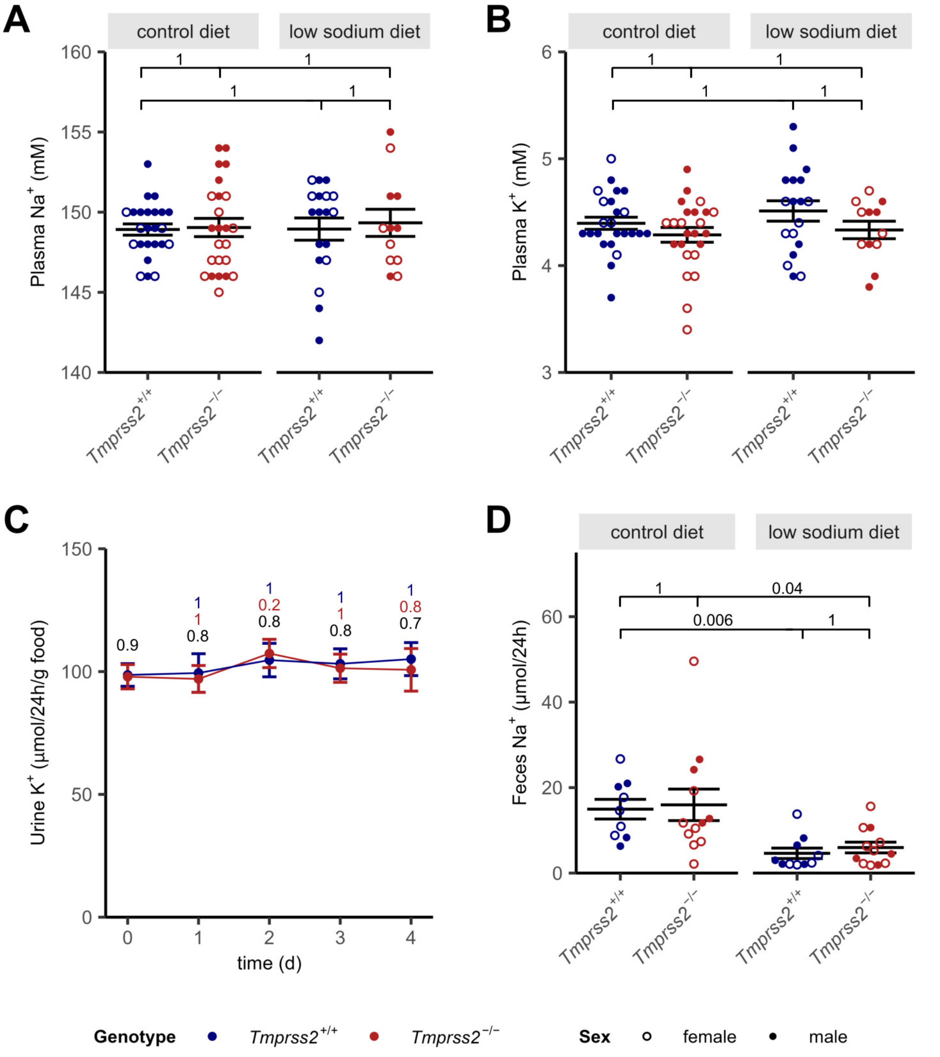

Supplemental Figure 17: Plasma Na^+ and K^+ concentrations, urinary K^+ excretion and fecal Na^+ excretion in response to dietary sodium restriction were not different in *Tmprss2*$^{-/-}$ compared to wildtype mice

(A-B, D) Plasma sodium (*A*) and potassium concentrations (*B*) and fecal sodium content (*D*) under control diet and after 4 days of low sodium diet. Data points from female and male mice are represented with open and closed symbols, respectively. Mean ± SEM and individual datapoints (n=12-24) are shown. Kruskal Wallis with Dunn's Multiple Comparisons Test.

(C) Timecourse of urinary potassium excretion, normalized to food intake, during dietary sodium restriction. Mean ± SEM (n=17-18) are shown. Data are pooled from both sexes. Red and blue *p*-values indicate comparisons to day 0 (Friedman test with Dunn's Multiple Comparison Test). Black *p*-values indicate comparisons between genotypes (Mann-Whitney Test).

Supplemental Figure 18

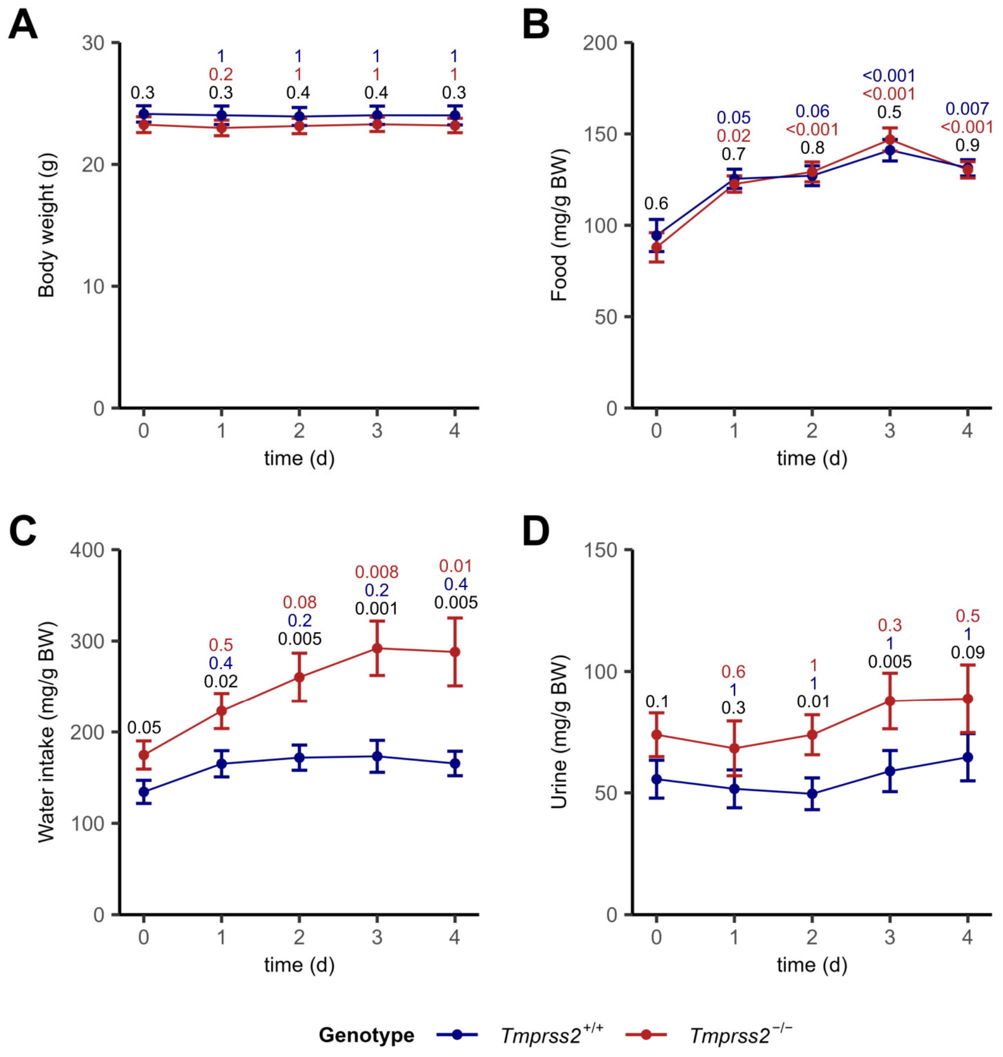

Supplemental Figure 18: Like *Tmprss2*$^{+/+}$ control mice, *Tmprss2*$^{-/-}$ mice maintained body weight under low sodium diet with similar food intake but higher water intake and urinary output

Timecourse of body weight (*A*), food intake (*B*), water intake (*C*), and urine output (*D*) during dietary sodium restriction. Values in (*B-D*) are normalized to body weight. Mean ± SEM (n=19-23) are shown. Red and blue *p*-values indicate comparisons to day 0 (*A, D*: Friedmann test with Dunn's Multiple Comparison Test; *B, C*: Kruskal-Wallis test with Dunn's Multiple Comparison Test). Black *p*-values indicate comparisons between genotypes (Mann-Whitney Test).

Supplemental Figure 19

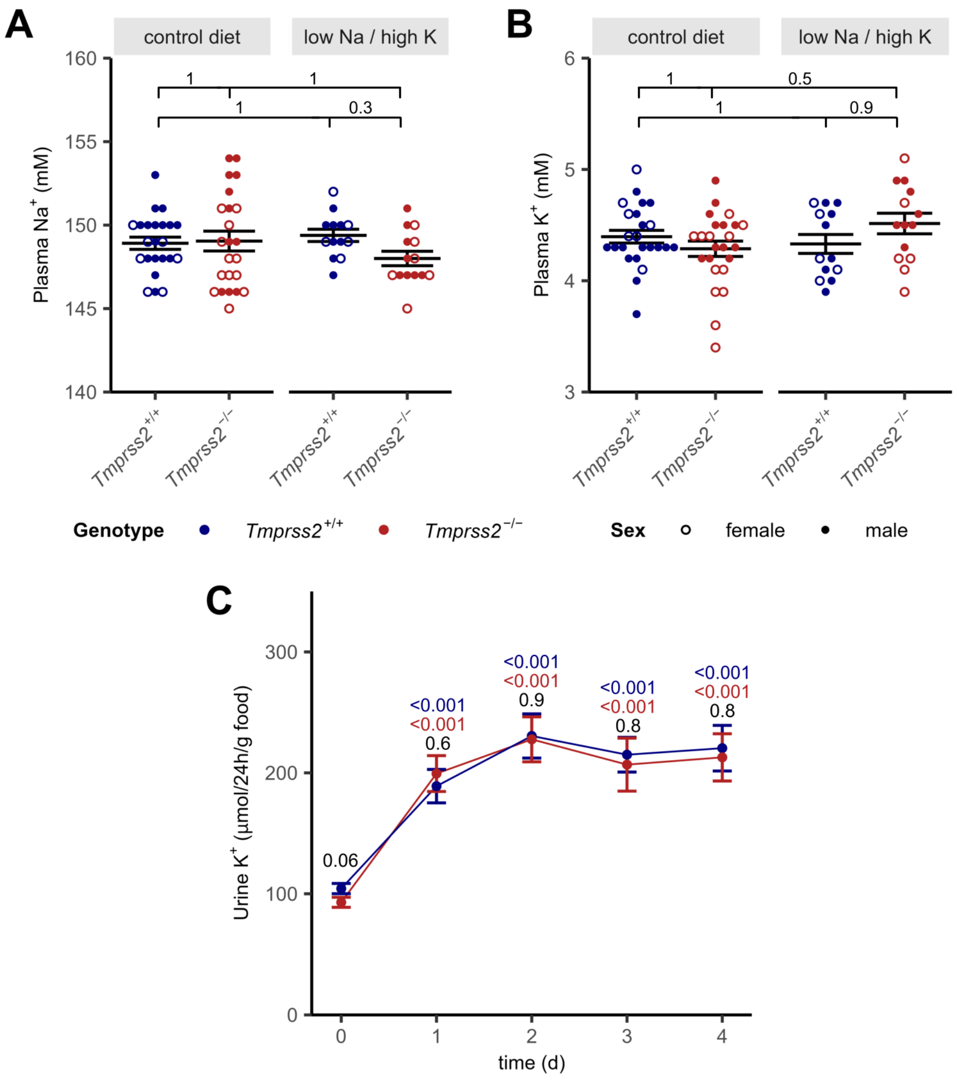

Supplemental Figure 19: In response to low sodium diet in combination with increased potassium intake, *Tmprss2$^{-/-}$* mice maintained sodium and potassium balance.

(A-B) Plasma sodium (*A*) and potassium (*B*) concentrations under control diet and after 4 days of low sodium diet with increased potassium intake. Data points from female and male mice are represented with open and closed symbols, respectively. Mean ± SEM and individual datapoints (n=13-23) are shown. Values for control diet are the same as in Supplemental Figure 17A, B.

(C) Timecourse of urinary potassium excretion, normalized to food intake, during dietary sodium restriction with increased potassium intake. Mean ± SEM (n=14-36) are shown. Data are pooled from both sexes. Red and blue *p*-values indicate comparisons to day 0 (Kruskal-Wallis with Dunn's Multiple Comparison Test). Black *p*-values indicate comparisons between genotypes (Mann-Whitney Test).

Supplemental Figure 20

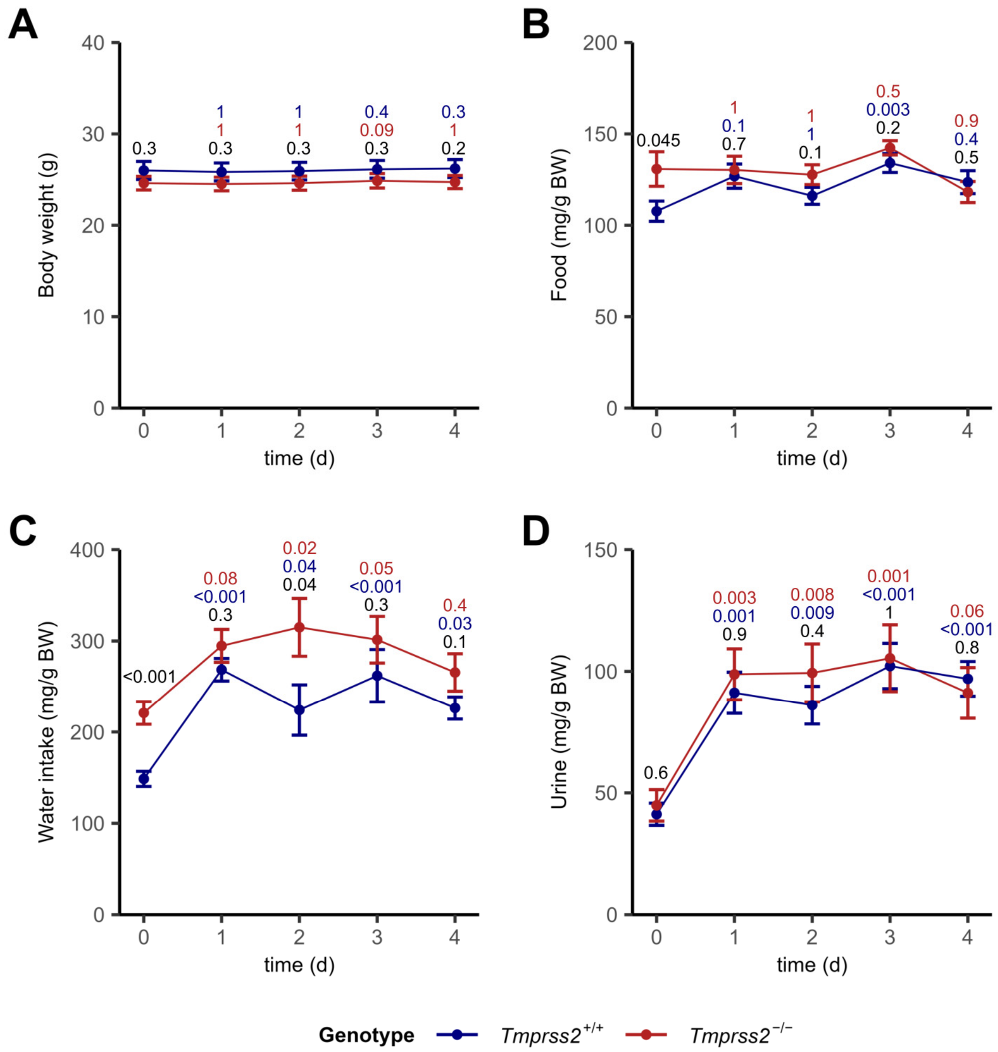

Supplemental Figure 20: Under low sodium diet in combination with increased potassium intake body weight, food intake, water intake and urine output were similar in *Tmprss2*$^{+/+}$ and *Tmprss2*$^{-/-}$ mice.

Timecourse of body weight (*A*), food intake (*B*), water intake (*C*), and urine output (*D*) during dietary sodium restriction in combination with increased potassium intake. Values in (*B-D*) are normalized to body weight. Mean ± SEM (n=13-14) are shown. Red and blue *p*-values indicate comparisons to day 0 (*A, B, D*: Friedmann test with Dunn's Multiple Comparison Test, *C*: Kruskal-Wallis test with Dunn's Multiple Comparison Test). Black *p*-values indicate comparisons between genotypes (Mann-Whitney Test).

Supplemental Figure 21

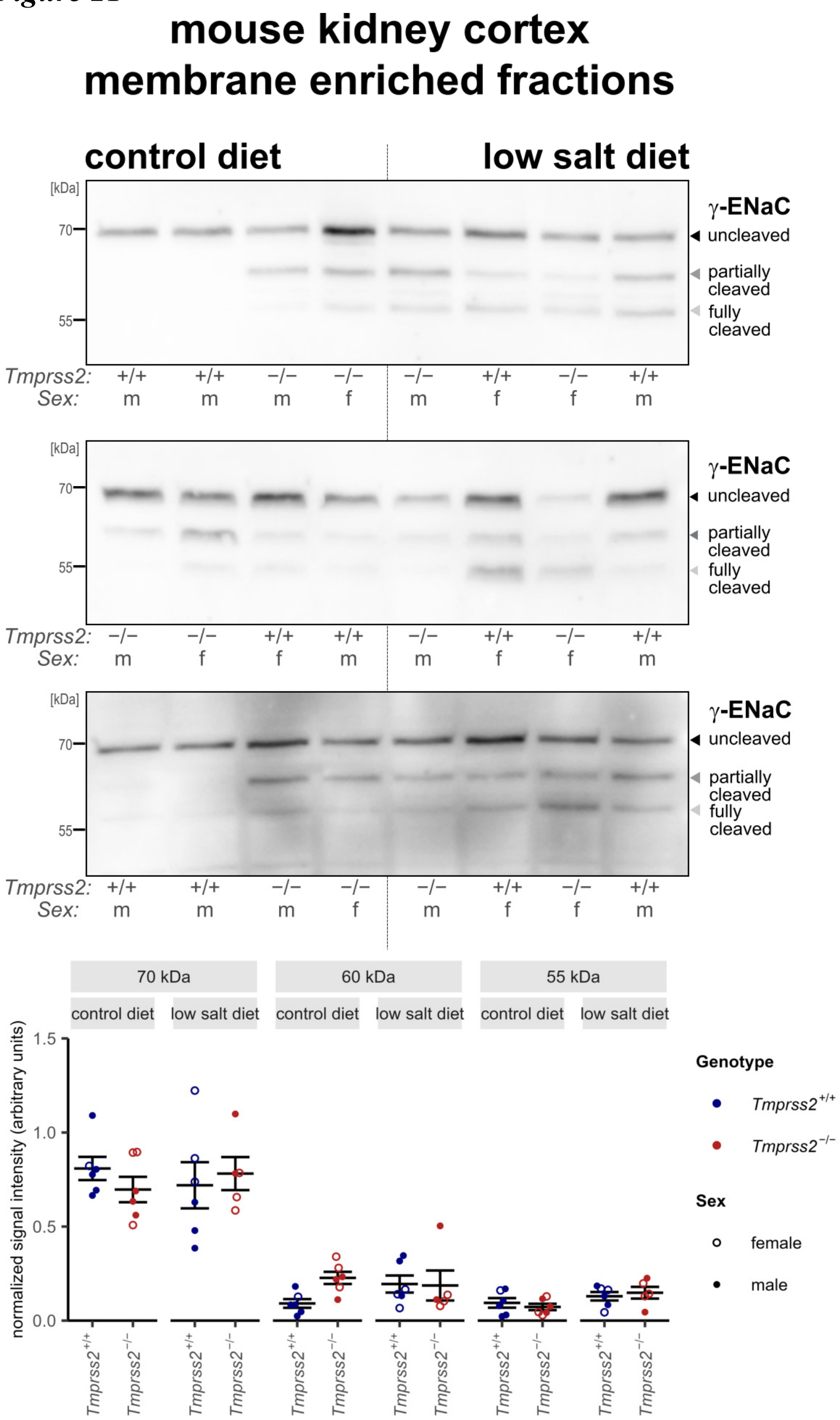

Supplemental Figure 21: Analysis of proteolytic processing of renal γ-ENaC under low salt diet.

(A) Western blots showing expression of γ-ENaC in membrane enriched fractions from mouse kidney cortex. Uncleaved (~70 kDa), partially cleaved (~60 kDa), and fully cleaved (~55 kDa) γ-ENaC are indicated with black, dark grey and light grey arrowheads, respectively. Kidneys were isolated after 4 days of control or low salt diet as indicated.

(B) Densitometric evaluation of similar western blots as in (*A*). The densitometric γ-ENaC signal in each lane was normalized to the Ponceau S total protein staining from the same lane. Mean ± SEM and data points for individual western blots are shown. Data points from female and male mice are represented with open and closed symbols, respectively. Please note that under control diet the abundance of the partially cleaved γ-ENaC fragment (60 kDa) appeared to be increased in *Tmprss2*$^{-/-}$ mice compared to wild-type mice consistent with the results shown in Figure 4 and S14. Unlike in Figure 4 this apparent trend did not reach statistical significance probably due to the smaller sample size and larger number of experimental groups included in multiple statistical testing. Kruskal-Wallis test (0.7 for 70 kDa fragment; 0.08 for 60 kDa fragment; 0.11 for 55 kDa fragment).

Supplemental Figure 22

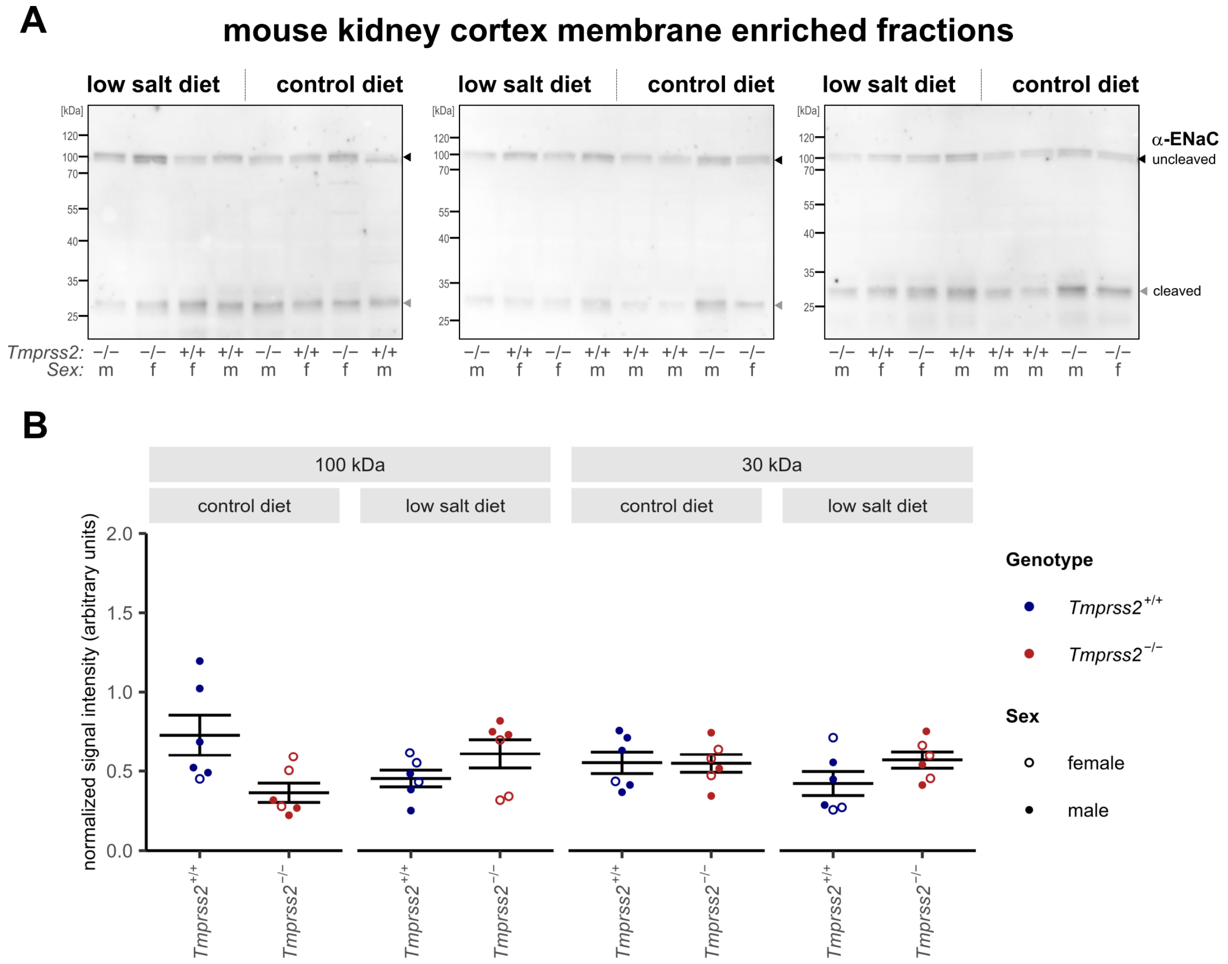

Supplemental Figure 22: Analysis of proteolytic processing of renal α-ENaC under low salt diet.

(A) Western blots showing expression of α-ENaC in membrane enriched fractions from mouse kidney cortex. Uncleaved (~100 kDa) and cleaved (~30 kDa) α-ENaC are indicated with a black and light grey arrowhead, respectively. Kidneys were isolated after 4 days of control or low salt diet as indicated.

(B) Densitometric evaluation of similar western blots as in (*A*). The densitometric α-ENaC signal in each lane was normalized to the Ponceau S total protein staining from the same lane. Mean ± SEM and data points for individual western blots are shown. Data points from female and male mice are represented with open and closed symbols, respectively. Kruskal-Wallis test (0.06 for 100 kDa fragment; 0.4 for 30 kDa fragment).

Supplemental Figure 23

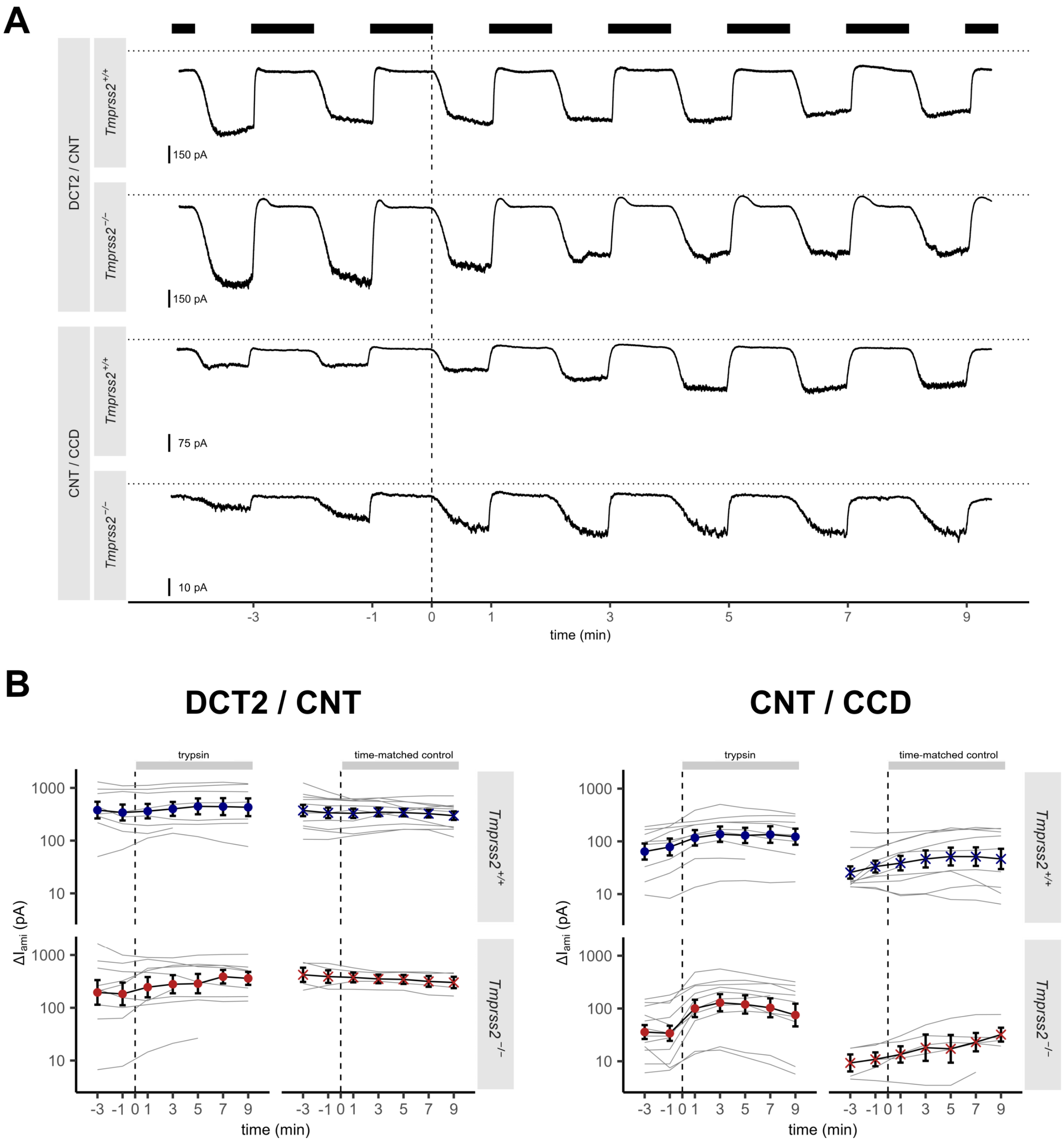

Supplemental Figure 23: Representative time-matched control recordings from microdissected tubules and summary of ΔI_{ami} time-courses in absolute values from individual experiments.

(A) Representative whole-cell current traces are shown from the DCT2/CNT and CNT/CCD isolated from *Tmprss2*$^{+/+}$ and *Tmprss2*$^{-/-}$ mice, as indicated. Presence of amiloride (4 µM) in the bath solution is indicated by black bars. All bath solutions contained 2 µg/ml soybean trypsin inhibitor (SBTI). A dotted line indicates zero current level. Timepoint of mock solution exchange, corresponding to the timepoint of trypsin application in Fig. 6A, is referred to as 0 min and marked with a dashed vertical line.

(B) Summary of absolute ΔI_{ami} values from similar experiments as shown in Figure 6A (trypsin, filled circles; first and third column) and from time-matched control recordings as shown in (*A*) (time-mached control, crosses; second and fourth column) displayed on a logarithmic scale. ΔI_{ami} was calculated as described in Figure 6B. Mean ± SEM are shown with symbols and error bars. Grey lines depict the timecourses of individual recordings. Corresponding normalized data are shown in Figure 6B.

Supplemental Tables

Supplemental Table 1

Significantly upregulated gene transcripts in $mCCD_{cl1}$ cells treated for 2 h with 3 nM aldosterone. Listed are all protein-coding transcripts with an at least 2-fold change in expression and an adjusted p-value < 0.05 (aldosterone-treated vs. vehicle-treated cells; n=6 per experimental group).

Gene name	Gene description	control average expression *(TPM)*			Fold change	adj. p-value
Sgk1	serum/glucocorticoid regulated kinase 1	25.92	±	2.42	8.99	<9E−99
Zbtb16	zinc finger and BTB domain containing 16	0.43	±	0.07	8.24	4E−98
Rasd1	RAS, dexamethasone-induced 1	0.83	±	0.08	3.01	1E−13
Gm49450	predicted gene, 49450	102.81	±	5.93	2.34	5E−02
Aldoart1	aldolase 1 A, retrogene 1	0.04	±	0.01	2.16	6E−03

Supplemental Table 2

Significantly up- and downregulated transcripts in $mCCD_{cl1}$ cells treated for 24 h with 3 nM aldosterone.

Listed are all protein-coding transcripts with an at least 2-fold change in expression and an adjusted p-value < 0.05 (aldosterone-treated vs. vehicle-treated cells; n=7 per experimental group). A dotted line separates upregulated from downregulated transcripts. **Klk1* (Kallikrein 1) has previously been proposed to play a role in ENaC regulation[27] [Patel AB, Chao J, Palmer LG. Tissue kallikrein activation of the epithelial Na^+ channel. *Am J Physiol Renal Physiol*. 2012; 303:F540-F550]. However, overall transcriptional expression of *Klk1* was very low compared to *Tmprss2* (~1 TPM versus >200 TPM).

Gene name	Gene description	control average expression *(TPM)*			Fold change	adj. p-value
Sgk1	serum/glucocorticoid regulated kinase 1	33.48	±	6.12	6.41	2E−16
Abcb5	ATP-binding cassette, sub-family B (MDR/TAP), member 5	0.13	±	0.04	4.59	7E−12
Sult1d1	sulfotransferase family 1D, member 1	10.09	±	1.21	3.32	4E−25
Slc2a9	solute carrier family 2 (facilitated glucose transporter), member 9	0.68	±	0.05	2.51	5E−17
Rasd1	RAS, dexamethasone-induced 1	2.03	±	0.13	2.41	3E−17
Aspg	asparaginase	0.20	±	0.04	2.39	3E−03
Zbtb16	zinc finger and BTB domain containing 16	1.81	±	0.37	2.35	7E−04
*Klk1**	kallikrein 1	0.76	±	0.05	2.20	8E−04
Defb2	defensin beta 2	10.12	±	1.25	2.13	1E−09
Apol8	apolipoprotein L 8	0.31	±	0.05	2.11	9E−04
Hspa1a	heat shock protein 1A	42.19	±	7.27	0.48	1E−02

Supplemental Table 3

Differentially expressed transcripts in TMRPSS2-ko *vs.* control mCCD$_{cl1}$ cells.

Listed are 20 protein-coding transcripts with the highest upregulation or downregulation in response to TMPRSS2-deficiency and an average expression in control cells of at least 1 TPM (Control *vs.* TMPRSS2-ko cells; n=6 per experimental group). A dotted line separates upregulated from downregulated transcripts.

Gene name	Gene description	control average expression *(TPM)*	Fold change	adj. p-value
Defb1	defensin beta 1	9.17 ± 1.50	3.24	2E−10
Nrep	neuronal regeneration related protein	4.24 ± 0.19	3.09	7E−10
Rasal1	RAS protein activator like 1 (GAP1 like)	1.53 ± 0.26	3.09	2E−31
Igfbp2	insulin-like growth factor binding protein 2	48.03 ± 5.24	3.05	1E−14
Hrct1	histidine rich carboxyl terminus 1	1.45 ± 0.19	3.04	2E−14
Megf6	multiple EGF-like-domains 6	3.02 ± 0.22	3.00	1E−12
Ggt1	gamma-glutamyltransferase 1	1.31 ± 0.16	2.87	6E−13
Arc	activity regulated cytoskeletal-associated protein	11.65 ± 0.90	2.80	3E−19
Lcn2	lipocalin 2	87.57 ± 5.59	2.80	3E−22
Cbr2	carbonyl reductase 2	19.78 ± 2.03	2.69	2E−16
Cpe	carboxypeptidase E	21.42 ± 0.96	2.68	5E−66
B3gnt7	UDP-GlcNAc:betaGal beta-1,3-N-acetylglucosaminyltransferase 7	2.77 ± 0.07	2.68	2E−29
Ntn4	netrin 4	22.31 ± 1.19	2.63	3E−33
Tgfb2	transforming growth factor, beta 2	12.19 ± 0.38	2.63	2E−79
Stc2	stanniocalcin 2	2.51 ± 0.10	2.62	1E−33
Elfn1	leucine rich repeat and fibronectin type III, extracellular 1	1.97 ± 0.17	2.62	1E−38
Isg15	ISG15 ubiquitin-like modifier	14.67 ± 3.74	2.61	2E−02
Ifit2	interferon-induced protein with tetratricopeptide repeats 2	12.54 ± 2.31	2.57	8E−04
Sgk1	serum/glucocorticoid regulated kinase 1	35.91 ± 4.84	2.57	8E−06
Ifit1	interferon-induced protein with tetratricopeptide repeats 1	19.15 ± 4.04	2.53	2E−02

Tmem52b	transmembrane protein 52B	1.26	±	0.12	0.27	3E−09
Tchh	trichohyalin	1.45	±	0.06	0.26	3E−36
Ces1d	carboxylesterase 1D	4.90	±	0.36	0.26	6E−34
Tmem229a	transmembrane protein 229A	1.25	±	0.08	0.25	7E−29
Ndn	necdin	2.45	±	0.10	0.25	2E−77
Klk8	kallikrein related-peptidase 8	1.41	±	0.18	0.24	1E−09
Eps8	epidermal growth factor receptor pathway substrate 8	1.37	±	0.16	0.24	5E−21
Lratd1	LRAT domain containing 1	1.30	±	0.06	0.23	2E−22
Aqp4	aquaporin 4	1.87	±	0.19	0.21	4E−31
Tanc2	tetratricopeptide repeat, ankyrin repeat and coiled-coil containing 2	1.58	±	0.15	0.21	9E−48
Gsdmc2	gasdermin C2	38.44	±	5.15	0.20	1E−16
Gsdmc3	gasdermin C3	14.38	±	1.98	0.20	5E−15
Cers6	ceramide synthase 6	1.23	±	0.03	0.20	5E−73
Pkib	protein kinase inhibitor beta, cAMP dependent, testis specific	3.27	±	0.38	0.19	3E−23
Htra1	HtrA serine peptidase 1	11.11	±	1.13	0.19	2E−58
St8sia4	ST8 alpha-N-acetyl-neuraminide alpha-2,8-sialyltransferase 4	3.03	±	0.31	0.19	3E−42
Col26a1	collagen, type XXVI, alpha 1	1.01	±	0.11	0.18	7E−17
Sprr2f	small proline-rich protein 2F	28.61	±	2.12	0.18	2E−71
Gsdmc4	gasdermin C4	2.60	±	0.39	0.15	2E−25
Npsr1	neuropeptide S receptor 1	1.91	±	0.28	0.11	1E−13
